# Natural family-free genomic distance

**DOI:** 10.1186/s13015-021-00183-8

**Published:** 2021-05-10

**Authors:** Diego P. Rubert, Fábio V. Martinez, Marília D. V. Braga

**Affiliations:** 1 Faculdade de Computação, Universidade Federal de Mato Grosso do Sul, Campo Grande, Brazil; 2 Faculty of Technology and Center for Biotechnology (CeBiTec), Bielefeld University, Bielefeld, Germany

**Keywords:** Comparative genomics, Genome rearrangement, DCJ-indel distance

## Abstract

**Background:**

A classical problem in comparative genomics is to compute the rearrangement distance, that is the minimum number of large-scale rearrangements required to transform a given genome into another given genome. The traditional approaches in this area are *family-based*, i.e., require the classification of DNA fragments of both genomes into *families*. Furthermore, the most elementary family-based models, which are able to compute distances in polynomial time, restrict the families to occur at most once in each genome. In contrast, the distance computation in models that allow multifamilies (i.e., families with multiple occurrences) is NP-hard. Very recently, Bohnenkämper et al. (J Comput Biol 28:410–431, 2021) proposed an ILP formulation for computing the genomic distance of genomes with multifamilies, allowing structural rearrangements, represented by the generic *double cut and join* (DCJ) operation, and content-modifying *insertions* and *deletions* of DNA segments. This ILP is very efficient, but must maximize a matching of the genes in each multifamily, in order to prevent the *free lunch* artifact that would otherwise let empty or almost empty matchings give smaller distances.

**Results:**

In this paper, we adopt the alternative *family-free* setting that, instead of family classification, simply uses the *pairwise similarities* between DNA fragments of both genomes to compute their rearrangement distance. We adapted the ILP mentioned above and developed a model in which pairwise similarities are used to assign weights to both matched and unmatched genes, so that an optimal solution does not necessarily maximize the matching. Our model then results in a *natural family-free genomic distance*, that takes into consideration all given genes, without prior classification into families, and has a search space composed of matchings of any size. In spite of its bigger search space, our ILP seems to be boosted by a reduction of the number of co-optimal solutions due to the weights. Indeed, it converged faster than the original one by Bohnenkämper et al. for instances with the same number of multiple connections. We can handle not only bacterial genomes, but also fungi and insects, or sets of chromosomes of mammals and plants. In a comparison study of six fruit fly genomes, we obtained accurate results.

**Supplementary Information:**

The online version contains supplementary material available at 10.1186/s13015-021-00183-8.

## Background

Genomes are subject to mutations or *rearrangements* in the course of evolution. A classical problem in comparative genomics is to compute the rearrangement *distance*, that is the minimum number of large-scale rearrangements required to transform a given genome into another given genome [1]. Typical large-scale rearrangements change the number of chromosomes, and/or the positions and orientations of DNA segments. Examples of such *structural* rearrangements are inversions, translocations, fusions and fissions. One might also need to consider rearrangements that modify the content of a genome, such as insertions and deletions (collectively called *indels*) of DNA segments.

In order to study the rearrangement distance, one usually adopts a high-level view of genomes, in which only “relevant” fragments of the DNA (e.g., genes) are taken into consideration. Furthermore, a pre-processing of the data is required, so that we can compare the content of the genomes. One popular method, adopted for more than 20 years, is to group the fragments in both genomes into *families*, so that two fragments in the same family are said to be equivalent. This setting is said to be *family-based*. Without duplications, that is, with the additional restriction that each family occurs at most once in each genome, many polynomial models have been proposed to compute the genomic distance [2–6]. However, when duplications are allowed the problem is more intricate and all approaches proposed so far are NP-hard, see for instance [7–12].

The required pre-classification of DNA fragments into families is a drawback of the family-based approaches. Moreover, even with a careful pre-processing, it is not always possible to classify each fragment unambiguously into a single family. Due to these facts, an alternative to the family-based setting was proposed and consists in studying the rearrangement distance without prior family assignment. Instead of families, the *pairwise similarities* between fragments is directly used [13, 14]. By letting structural rearrangements be represented by the generic *double cut and join* (DCJ) operation [4], a first family-free genomic distance, called family-free DCJ distance, was already proposed [15]. Its computation helps to match occurrences of duplicated genes and find homologies, but unmatched genes are simply ignored.

In the family-based setting, the mentioned approaches that handle duplications either require the compared genomes to be *balanced* (that is, have the same number of occurrences of each family) [11, 12] or adopt some approach to match genes, ignoring unmatched genes [7, 9]. Recently, a new family-based approach was proposed, allowing each family to occur any number of times in each genome and integrating DCJ operations and indels in a *DCJ-indel* distance formula [16]. For its computation, that is NP-hard, an efficient ILP was proposed.

Here we adapt the approach mentioned above and give an ILP formulation to compute a new family-free DCJ-indel distance. In the family-based approach from [16] as well as in the family-free DCJ distance proposed in [15], the search space needs to be restricted to candidates that maximize the number of matched genes, in order to avoid the *free lunch* artifact that would otherwise let empty or almost empty matchings give smaller distances [5]. In our formulation we use the pairwise similarities to assign weights to matched and unmatched genes, so that, for the first time, an optimal solution does not necessarily maximize the number of matched genes. For the fact that our model takes into consideration all given genes and has a search space composed of matchings of any size, we call it *natural family-free genomic distance*. Our simulated experiments show that our ILP can handle not only bacterial genomes, but also complete genomes of fungi and insects, or sets of chromosomes of mammals and plants. We use our implementation to generate pairwise distances and reconstruct the phylogeny of six species of fruit flies from the genus *Drosophila*, obtaining accurate results.

This paper is an extended version of a work presented at WABI 2020 [17].

## Preliminaries

We call *marker* an oriented DNA fragment. A *chromosome* is composed of markers and can be linear or circular. A marker *m* in a chromosome can be represented by the symbol *m* itself, if it is read in direct orientation, or the symbol $\overline{m}$, if it is read in reverse orientation. We concatenate all markers of a chromosome *Z* in a string *z*, which can be read in any of the two directions. If *Z* is linear, the string *z* is flanked by square brackets. If *Z* is circular, we can start to read it at any marker and the string *z* is flanked by parentheses. A set of chromosomes comprises a *genome*. As an example, let $A=\{\,[\,{\overline{\mathtt{6}}}\,{\mathtt{1}}\,{\mathtt{7}}\,{\mathtt{8}}\,{\overline{\mathtt{4}}}\,], [\,{\mathtt{3}}\,{\overline{\mathtt{5}}}\,{\mathtt{2}}\,] \,\}$ be a genome composed of two linear chromosomes. A genome can be transformed or *sorted* into another genome with the following types of mutations.

1. DCJ operations modify the organization of a genome: A *cut* performed on a genome *A* separates two adjacent markers of *A*. A *double-cut and join* or *DCJ* applied on a genome *A* is the operation that performs cuts in two different positions of *A*, creating four open ends, and joins these open ends in a different way [2, 4]. For example, let $A=\{\,[\,\overline{\mathtt{6}}\,\mathtt{1}\,\mathtt{7}\,\mathtt{8}\,\overline{\mathtt{4}}\,], [\,\mathtt{3}\,\overline{\mathtt{5}}\,\mathtt{2}\,]\,\}$, and consider a DCJ that cuts between markers $\mathtt{1}$ and $\mathtt{7}$ of its first chromosome and between markers $\mathtt{5}$ and $\mathtt{2}$ of its second chromosome, creating segments $\overline{\mathtt{6}}\,\mathtt{1}\bullet$, $\bullet \mathtt{7}\,\mathtt{8}\,\overline{\mathtt{4}}$, $\mathtt{3}\,\overline{\mathtt{5}}\bullet$ and $\bullet \mathtt{2}$ (where the symbols $\bullet$ represent the open ends). If we join the first with the fourth and the second with the third open end, we get $A'=\{\,[\,\overline{\mathtt{6}}\,\mathtt{1}\,\mathtt{2}\,], [\,\mathtt{3}\,\overline{\mathtt{5}}\,\mathtt{7}\,\mathtt{8}\,\overline{\mathtt{4}}\,]\,\}$, that is, the described DCJ operation is a translocation transforming *A* into $A'$. Indeed, a DCJ operation can correspond not only to a translocation but to several structural rearrangements, such as an inversion, a fusion or a fission. (Note that a DCJ is a symmetric operation: in the example above, we can transform $A'$ into *A* with a DCJ operation whose cuts create the same open segments $\overline{\mathtt{6}}\,\mathtt{1}\bullet$, $\bullet \mathtt{2}$, $\mathtt{3}\,\overline{\mathtt{5}}\bullet$ and  $\bullet \mathtt{7}\,\mathtt{8}\,\overline{\mathtt{4}}$.)
2. Indel operations modify the content of a genome: The content of a genome can be modified with *insertions* and with *deletions* of blocks of contiguous markers, collectively called *indel* operations [5, 6]. As an example, consider the deletion of segment $\mathtt{7}\,\mathtt{8}$ from chromosome $[\,\overline{\mathtt{6}}\,\mathtt{1}\,\mathtt{7}\,\mathtt{8}\,\overline{\mathtt{4}}\,]$, resulting in chromosome $[\,\overline{\mathtt{6}}\,\mathtt{1}\,\overline{\mathtt{4}}\,]$. (An indel operation is also symmetric: the inverse of the given example would be the insertion of segment $\mathtt{7}\,\mathtt{8}$ between markers $\mathtt{1}$ and $\mathtt{4}$ in chromosome $[\,\overline{\mathtt{6}}\,\mathtt{1}\,\overline{\mathtt{4}}\,]$, resulting in $[\,\overline{\mathtt{6}}\,\mathtt{1}\,\mathtt{7}\,\mathtt{8}\,\overline{\mathtt{4}}\,]$). In the model we consider, we do not allow that a marker is deleted and reinserted, nor inserted and then deleted. Furthermore, at most one chromosome can be entirely deleted or inserted at once. In the comparison of two genomes, these restrictions prevent the *free lunch* artifact of sorting one genome into the other by simply deleting the content of the first and inserting the content of the second, ignoring their common parts, but does not guarantee that distances including indel operations are metric. Indeed, indel operations allow comparisons of genomes of very distinct contents and sizes and may disrupt the triangular inequality [18].

The *DCJ-indel distance* of two genomes *A* and *B* is the minimum number of DCJ and indel operations required to transform *A* into *B* (or *vice-versa*). Denote by ${\mathcal {G}}(A)$ the set of markers in genome *A* and by ${\mathcal {G}}(B)$ the set of markers in genome *B*. In the present work we consider two distinct settings:

- In a *family-based setting* markers are grouped into families. Let ${\mathcal {F}}(A)$ be the set of families in genome *A* and ${\mathcal {F}}(B)$ be the set of families in genome *B*.
  Each marker from a genome is represented by its family, and a family can occur more than once in each genome, i. e., here the sets ${\mathcal {G}}(A)$ and  ${\mathcal {G}}(B)$ are multisets that may contain more than one copy of each marker. Genomes *A* and *B* may share a set of *common families* ${\mathcal {F}}_{\star }= {\mathcal {F}}(A) \cap {\mathcal {F}}(B)$. We also have sets ${\mathcal {A}}= {\mathcal {F}}(A) \setminus {\mathcal {F}}_{\star }$ and ${\mathcal {B}}= {\mathcal {F}}(B) \setminus {\mathcal {F}}_{\star }$ of families that occur respectively only in *A* and only in *B* and are called *exclusive families*. Markers from exclusive families are called *exclusive markers*. A family that occurs at most once in each genome is said to be *singular*. For example, we could have $A=\{\,[\,\overline{\mathtt{3}}\,\mathtt{1}\,\mathtt{4}\,\mathtt{3}\,\overline{\mathtt{2}}\,], [\,\mathtt{3}\,\overline{\mathtt{5}}\,\mathtt{2}\,]\,\}$ and $B=\{\,[\,\overline{\mathtt{1}}\,\mathtt{2}\,\overline{\mathtt{3}}\,\mathtt{3}\,\overline{\mathtt{2}}\,\mathtt{6}\,]\,\}$. In this case we have ${\mathcal {F}}(A) = \{\mathtt{1},\mathtt{2},\mathtt{3},\mathtt{4},\mathtt{5}\}$ and ${\mathcal {F}}(B) = \{\mathtt{1},\mathtt{2},\mathtt{3},\mathtt{6}\}$. Consequently, ${\mathcal {F}}_{\star }= \{\mathtt{1},\mathtt{2},\mathtt{3}\}$, ${\mathcal {A}}= \{\mathtt{4},\mathtt{5}\}$ and ${\mathcal {B}}= \{\mathtt{6}\}$. Note also that ${\mathcal {G}}(A) = \{\mathtt{1},\mathtt{2},\mathtt{2},\mathtt{3},\mathtt{3},\mathtt{3},\mathtt{4},\mathtt{5}\}$ and ${\mathcal {G}}(B) = \{\mathtt{1},\mathtt{2},\mathtt{2},\mathtt{3},\mathtt{3},\mathtt{6}\}$. Here the set of singular families is $\{1,4,5,6\}$.
- In a *family-free setting* the markers of *A* and *B* are all distinct and unique. In other words, sets ${\mathcal {G}}(A)$ and ${\mathcal {G}}(B)$ are necessarily simple sets, and ${\mathcal {G}}(A) \cap {\mathcal {G}}(B)=\emptyset$. An example here is the pair of genomes $A=\{\,[\,\overline{\mathtt{1}}\,\mathtt{3}\,\overline{\mathtt{4}}\,\mathtt{2}\,]\,\}$ and $B=\{\,[\,\overline{\mathtt{8}}\,\mathtt{7}\,\overline{\mathtt{5}}\,], [\,\mathtt{9}\,\overline{\mathtt{6}}\,]\,\}$, with ${\mathcal {G}}(A) = \{\mathtt{1},\mathtt{2},\mathtt{3},\mathtt{4}\}$ and ${\mathcal {G}}(B) = \{\mathtt{5},\mathtt{6},\mathtt{7},\mathtt{8},\mathtt{9}\}$.

### Relational diagram and DCJ-indel distance of family-based singular genomes

Let *A* and *B* be two genomes in a family-based setting and assume that both *A* and *B* are *singular*, that is, each common family from ${\mathcal {F}}_{\star }= {\mathcal {F}}(A) \cap {\mathcal {F}}(B)$ is singular, occurring exactly once in each genome.^1^ We will now describe how the DCJ-indel distance can be computed in this case [6].

For a given marker *m*, denote its two extremities by $m^{\,\!t}$ (tail) and $m^{\,\!h}$ (head). Given two singular genomes *A* and *B*, the *relational diagram* *R*(*A*, *B*) [16] has a set of vertices $V = V(A) \cup V(B)$, where *V*(*A*) is the set of extremities of markers from *A* and *V*(*B*) is the set of extremities of markers from *B*. There are three types of edges in *R*(*A*, *B*):

- *Adjacency edges*: for each pair of marker extremities $\gamma _1$ and $\gamma _2$ that are adjacent in a chromosome of any of the two genomes, we have the adjacency edge $\gamma _1\gamma _2$. Denote by $E_{\text {adj}}^{A}$ and by $E_{\text {adj}}^{B}$ the adjacency edges in *A* and in *B*, respectively. Marker extremities located at chromosome ends are called *telomeres* and are not connected to any adjacency edge.
- *Extremity edges*, whose set is denoted by $E_{\gamma }$: for each common family $m\in {\mathcal {F}}_{\star }$, we have two extremity edges, one connecting the vertex $m^{\,\!h}$ from *V*(*A*) to the vertex $m^{\,\!h}$ from *V*(*B*) and the other connecting the vertex $m^{\,\!t}$ from *V*(*A*) to the vertex $m^{\,\!t}$ from *V*(*B*).
- *Indel edges*: for each occurrence of an exclusive family $m\in {\mathcal {A}}\cup {\mathcal {B}}$, we have the indel edge $m^{\,\!t}m^{\,\!h}$. Denote by $E_{\text {id}}^{A}$ and by $E_{\text {id}}^{B}$ the indel edges in *A* and in *B*, respectively.

**Fig. 1:**
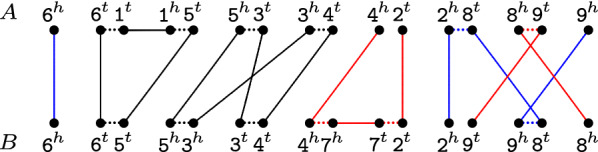
Relational diagram of two singular genomes. For genomes $A=\{\,[\,\overline{\mathtt{6}}\,\mathtt{1}\,\mathtt{5}\,\mathtt{3}\,\mathtt{4}\,], [\,\mathtt{2}\,\mathtt{8}\,\mathtt{9}\,]\,\}$ and $B=\{\,[\,\overline{\mathtt{6}}\,\mathtt{5}\,\overline{\mathtt{3}}\,\mathtt{4}\,\overline{\mathtt{7}}\,\mathtt{2}\,], [\,\mathtt{9}\,\mathtt{8}\,]\,\}$, the relational diagram contains two cycles, two $AB$-paths (represented in blue), one $AA$-path and one $BB$-path (both represented in red). Short dotted horizontal edges are adjacency edges, long horizontal edges are indel edges, top-down edges are extremity edges

Each vertex has degree one or two: it is connected either to an extremity edge or to an indel edge, and to at most one adjacency edge, therefore *R*(*A*, *B*) is a simple collection of cycles and paths. A path that has one endpoint in genome *A* and the other in genome *B* is called an $AB$-*path*. In the same way, both endpoints of an $AA$-*path* are in *A* and both endpoints of a $BB$-*path* are in *B*. A cycle contains either zero or an even number of extremity edges. When a cycle has at least two extremity edges, it is called an $AB$-*cycle*. Moreover, a path (respectively cycle) of *R*(*A*, *B*) composed exclusively of indel and adjacency edges in one of the two genomes corresponds to a whole linear (respectively circular) chromosome and is called a *linear* (respectively *circular*) *singleton* in that genome. Actually, linear singletons are particular cases of $AA$- or $BB$-paths. Since there is an even number of telomeres in *R*(*A*, *B*), the number of $AB$-paths is always even. An example of a relational diagram is given in Fig. 1.

#### DCJ distance of canonical genomes

When singular genomes *A* and *B* have no exclusive families, that is, ${\mathcal {A}}= {\mathcal {B}}=\emptyset$, they are said to be *canonical*. In this case *A* can be sorted into *B* with DCJ operations only and their DCJ distance $d_{DCJ}$ can be computed as follows [2]:

$$\begin{aligned} d_{DCJ} (A,B) \;=\; |{\mathcal {F}}_{\star }| - c - \frac{i}{2}\,, \end{aligned}$$

where *c* is the number of $AB$-cycles and *i* is the number of $AB$-paths in *R*(*A*, *B*).

#### Runs and indel-potential

When singular genomes *A* and *B* have exclusive families, it is possible to optimally select DCJ operations that group exclusive markers together for minimizing indels [6], as follows.

Given two genomes *A* and *B* and a component *C* of *R*(*A*, *B*), a *run* [6] is a maximal subpath of *C*, in which the first and the last edges are indel edges, and all indel edges belong to the same genome. It can be an ${\mathcal {A}}$-run when its indel edges are in genome *A*, or a ${\mathcal {B}}$-run when its indel edges are in genome *B*. We denote by $\Lambda (C)$ the number of runs in component *C*. If $\Lambda (C)\ge 1$ the component *C* is said to be *indel-enclosing*, otherwise $\Lambda (C)=0$ and *C* is said to be *indel-free*. The *indel-potential* of a component *C*, denoted by $\lambda (C)$, is the optimal number of indels obtained after “sorting” *C* separately and can be directly computed from $\Lambda (C)$ [6]:

$$\begin{aligned} \lambda (C) = {\left\{ \begin{array}{ll} ~~~~~0\,, &{} \text{ if } \Lambda (C)=0~~~(C \text{ is } \text{ indel-free) };\\ \left\lceil \frac{\Lambda (C)+1}{2}\right\rceil \,, &{} \text{ if } \Lambda (C) \ge 1~~~(C \text{ is } \text{ indel-enclosing) }. \end{array}\right. } \end{aligned}$$

An illustration of a $BB$-path with 4 runs and how its indel-potential can be achieved is given in Additional file 1: Figure S1-1, Appendix S1, Section (1A). With the indel-potential, an upper bound for the DCJ-indel distance $d_{DCJ}^{~id}$ was established [6]:

1 $$\begin{aligned} d_{DCJ}^{~id} (A,B) \;\le \; |{\mathcal {F}}_{\star }| - c - \frac{i}{2}~+ \sum _{C \in R(A,B)} \lambda (C) \end{aligned}$$

#### DCJ-indel distance of singular circular genomes

For singular circular genomes, the graph *R*(*A*, *B*) is composed of cycles only. In this case the upper bound given by Eq. (1) is tight and leads to a simplified formula [6]:

$$\begin{aligned} d_{DCJ}^{~id} (A,B) \;=\; |{\mathcal {F}}_{\star }| - c~+ \sum _{C \in R(A,B)} \lambda (C). \end{aligned}$$

#### DCJ-indel distance of singular linear genomes

For singular linear genomes, the upper bound given by Eq. (1) is achieved when the components of *R*(*A*, *B*) are sorted separately. However, it can be decreased by *recombinations*, that are DCJ operations that act on two distinct paths of *R*(*A*, *B*). Such path recombinations are said to be *deducting*. The total number of types of deducting recombinations is relatively small. By exhaustively exploring the space of recombination types, it is possible to identify groups of chained recombinations [6], so that the sources of each group are the original paths of the graph. In other words, a path that is a resultant of a group is never a source of another group. This results in a greedy approach (detailed in [6]) that optimally finds the value $\delta \ge 0$ to be deducted. We then have the following exact formula [6]:

$$\begin{aligned} d_{DCJ}^{~id} (A,B) \;=\; |{\mathcal {F}}_{\star }| - c - \frac{i}{2}~+ \sum _{C \in R(A,B)} \lambda (C) ~~- \delta . \end{aligned}$$

### DCJ-indel distance of family-based natural genomes

Two genomes *A* and *B* in a family-based setting are said to be *natural* when no restriction on the number of occurrences of each family in each genome is imposed. An approach to compute the DCJ-indel distance of natural genomes was proposed recently by Bohnenkämper et al. [16] and is briefly described below.

Given a family $f \in {\mathcal {F}}_{\star }$, let $\Phi _{A}(f)$ be the number of occurrences of *f* in genome *A* and $\Phi _{B}(f)$ be the number of occurrences of *f* in genome *B*. A common family whose number of occurrences is bigger than one in at least one of the two genomes is called a *multifamily*. Natural genomes *A* and *B* can be transformed into *linked* singular genomes $A^{\ddagger }$ and $B^{\ddagger }$ by disambiguating all multifamilies: for each multifamily *f*, a maximum set of one-to-one correspondences between occurrences of *f* in *A* and in *B* has to be established. The pairs of corresponding occurrences are then called *linked occurrences*. Since the disambiguation maximizes the number of linked occurrences, for each multifamily *f* in each genome, this number is $\min \{\Phi _{A}(f),\Phi _{B}(f)\}$. The linked occurrences are assumed to belong to the same new singular family and receive the same identifier in $A^{\ddagger }$ and in $B^{\ddagger }$ (e.g., by having the same *index* assigned). For example, many distinct pairs of linked singular genomes can be derived from natural genomes $A=[\,\mathtt{1}\,\,\mathtt{3}\,\,\overline{\mathtt{5}}\,\,\overline{\mathtt{2}}\,\,\mathtt{3}\,\,\mathtt{5}\,\,\mathtt{2}\,]$ and $B=[\,\mathtt{1}\,\,\mathtt{3}\,\,\mathtt{1}\,\,\mathtt{6}\,\,\mathtt{3}\,\,\mathtt{2}\,\,\mathtt{1}\,\,\mathtt{3}\,]$, including:

$$\begin{aligned} A^{\ddagger _{1}}= & {} [\,\mathtt{1}_{{\mathtt{1}}}\,\,\mathtt{3}_{{\mathtt{1}}}\,\,\overline{\mathtt{5}}\,\,\mathtt{\overline{2}}_{{\mathtt{1}}}\,\,\mathtt{3}_{{\mathtt{2}}}\,\,\mathtt{5}\,\,\mathtt{2}\,]~,~~ B^{\ddagger _{1}}=[\,\mathtt{1}\,\,\mathtt{3}_{{\mathtt{1}}}\,\,\mathtt{1}_{{\mathtt{1}}}\,\,\mathtt{6}\,\,\mathtt{3}_{{\mathtt{2}}}\,\,\mathtt{2}_{{\mathtt{1}}}\,\,\mathtt{1}\,\,\mathtt{3}\,]\,,~~\hbox {and}\\ A^{\ddagger _{2}}= & {} [\,\mathtt{1}_{{\mathtt{1}}}\,\,\mathtt{3}_{{\mathtt{1}}}\,\,\overline{\mathtt{5}}\,\,\overline{\mathtt{2}}\,\,\mathtt{3}_{{\mathtt{2}}}\,\,\mathtt{5}\,\,\mathtt{2}_{{\mathtt{1}}}\,]~,~~ B^{\ddagger _{2}}=[\,\mathtt{1}_{{\mathtt{1}}}\,\,\mathtt{3}_{{\mathtt{2}}}\,\,\mathtt{1}\,\,\mathtt{6}\,\,\mathtt{3}_{{\mathtt{1}}}\,\,\mathtt{2}_{{\mathtt{1}}}\,\,\mathtt{1}\,\,\mathtt{3}\,]. \end{aligned}$$

The DCJ-indel distance $nd_{DCJ}^{~id}$ of natural genomes *A* and *B* is then defined as

$$\begin{aligned} nd_{DCJ}^{~id} (A, B) = \min _{(A^{\ddagger },B^{\ddagger }) \in {\mathbb {X}}}\{ d_{DCJ}^{~id} (A^{\ddagger },B^{\ddagger }) \} \,, \end{aligned}$$

where ${\mathbb {X}}$ is the set of all possible pairs of linked singular genomes derived from natural genomes *A* and *B*. Computing $nd_{DCJ}^{~id} (A,B)$ is an NP-hard problem, and an ILP formulation to solve it was provided in [16].

## The family-free setting

As already stated, in the family-free setting, each marker in each genome is represented by a distinct symbol, therefore ${\mathcal {G}}(A)$ and ${\mathcal {G}}(B)$ are simple sets, and additionally ${\mathcal {G}}(A) \cap {\mathcal {G}}(B) = \emptyset$. Observe that the cardinalities $|{\mathcal {G}}(A)|$ and $|{\mathcal {G}}(B)|$ may be distinct.

### Marker similarity graph for the family-free setting

**Fig. 2:**
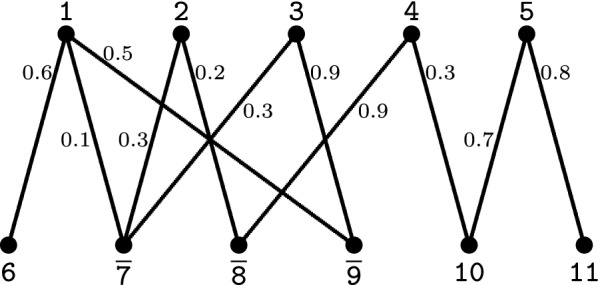
Example of similarity graph. Here we show the graph ${\mathcal {S}}_{0.1}(A,B)$ for the two genomes $A=\{\,[\,\mathtt{1}\,\,\mathtt{2}\,\,\mathtt{3}\,\,\mathtt{4}\,\,\mathtt{5}\,]\,\}$ and $B=\{\,[\,\mathtt{6}\,\,\overline{\mathtt{7}}\,\,\overline{\mathtt{8}}\,\,\overline{\mathtt{9}}\,\,\mathtt{10}\,\,\mathtt{11}\,]\,\}$

Given a threshold $0 \le x \le 1$, we can represent the similarities between the markers of genome *A* and the markers of genome *B* in the so called *marker similarity graph* [14], denoted by ${\mathcal {S}}_{x}(A, B)$. This is a weighted bipartite graph whose partitions ${\mathcal {G}}(A)$ and ${\mathcal {G}}(B)$ are the sets of markers in genomes *A* and *B*, respectively. Furthermore, for each pair of markers $a\in {\mathcal {G}}(A)$ and $b\in {\mathcal {G}}(B)$, denote by $\sigma (a, b)$ their *normalized similarity*, a value that ranges in the interval [0, 1]. If $\sigma (a, b) \ge x$ there is an edge *e* connecting *a* and *b* in ${\mathcal {S}}_{x}(A,B)$ whose weight is $\sigma (e) := \sigma (a, b)$. An example is given in Fig. 2.

#### Mapped family-based singular genomes

Let *A* and *B* be two family-free genomes with marker similarity graph ${\mathcal {S}}_{x}(A,B)$ and let $M = \{e_1, e_2, \ldots , e_n\}$ be a matching in ${\mathcal {S}}_{x}(A,B)$. Since the endpoints of each edge $e_i = (a, b)$ in *M* are not saturated by any other edge of *M*, we can unambiguously define the function $s(a,M) = s(b,M) = i$. We then define the set of *M*-*saturated mapped families*:

$$\begin{aligned} {\mathcal {F}}_{\star }(M)&= \{ s(g,M) :g \text{ is } M \text{-saturated}\}\\&=\{1,2,\ldots ,n\}.\end{aligned}$$

Let ${\tilde{n}}_A$ be the number of unsaturated markers in ${\mathcal {A}}$ and ${\tilde{n}}_B$ be the number of unsaturated markers in ${\mathcal {B}}$. We extend the function *s*, so that it maps each unsaturated marker $a'\in {\mathcal {A}}$ to one value in $\{n+1, n+2, \ldots , n+{\tilde{n}}_A\}$ and each unsaturated marker $b' \in {\mathcal {B}}$ to one value in $\{n+{\tilde{n}}_A+1, n+{\tilde{n}}_A+2, \ldots , n+{\tilde{n}}_A+{\tilde{n}}_B\}$. The sets of *M*-*unsaturated mapped families* are:

$$\begin{aligned} {\mathcal {A}}(M)&= \{ s(a',M) :a' \in {\mathcal {A}} \text{ is } M \text{-unsaturated}\}\\&=\{n+1,n+2,\ldots ,n+{\tilde{n}}_A\} \end{aligned}$$

and

$$\begin{aligned} {\mathcal {B}}(M)&= \{ s(b',M) :b' \in {\mathcal {B}} \text{ is } M \text{-unsaturated}\}\\&=\{n+{\tilde{n}}_A+1,n+{\tilde{n}}_A+2,\ldots ,n+{\tilde{n}}_A+{\tilde{n}}_B\}. \end{aligned}$$

The *mapped family-based singular genomes* $A^M$ and $B^M$ are then obtained by renaming each marker $a \in {\mathcal {A}}$ to *s*(*a*, *M*) and each marker $b\in {\mathcal {B}}$ to *s*(*b*, *M*), preserving all orientations.

#### Established distances of mapped family-based singular genomes

Let the relational diagram $R(A^M,B^M)$ have $c_M$ $AB$-cycles and $i_M$ $AB$-paths and note that $|{\mathcal {F}}_{\star }(M)|=|M|$. By simply ignoring the exclusive markers of families ${\mathcal {A}}(M)$ and ${\mathcal {B}}(M)$, we can compute the DCJ distance:

$$\begin{aligned} d_{DCJ} (A^M, B^M) = |M| -c_M -\frac{i_M}{2}. \end{aligned}$$

Taking into consideration the weight of the matching *M* defined as $w(M) = \sum _{e \in M} \sigma (e)$, we can also compute the weighted DCJ distance $wd_{DCJ}(A^M, B^M)$ [15]:

$$\begin{aligned} wd_{DCJ}(A^M, B^M) = d_{DCJ} (A^M, B^M) + |M| - w(M). \end{aligned}$$

Observe that, when all edges of *M* have the maximum weight 1, we have $w(M)=|M|$ and $wd_{DCJ}(A^M, B^M) = d_{DCJ} (A^M, B^M)$.

Finally, taking into consideration the markers from exclusive families ${\mathcal {A}}(M)$ and ${\mathcal {B}}(M)$, but not the weight *w*(*M*), we can compute the DCJ-indel distance of mapped genomes $A^M$ and $B^M$:

$$\begin{aligned} d_{DCJ}^{~id} (A^M,B^M)&= |M| -c_M -\frac{i_M}{2} + \sum \limits _{{C \in R(A^M,B^M)}} \lambda (C) ~- \delta _M\,, \end{aligned}$$

where $\delta _M$ is the deduction given by path recombinations in $R(A^M,B^M)$.

### The family-free DCJ-indel distance

**Fig. 3:**
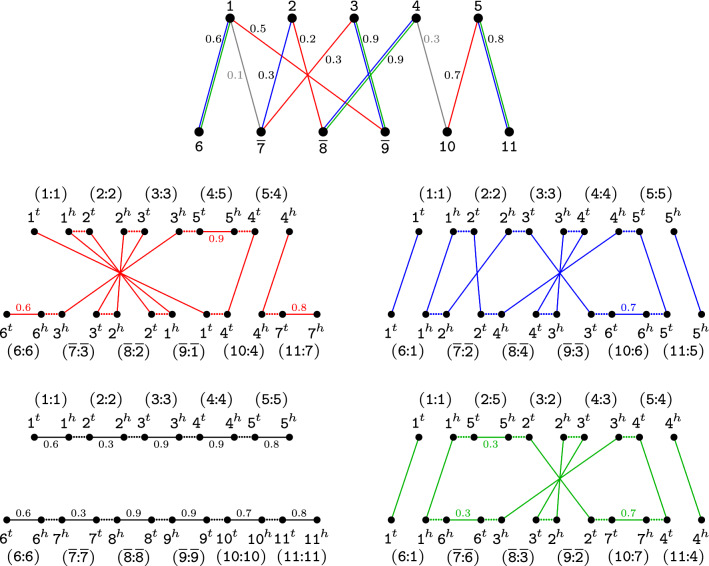
Matchings of a similarity graph and their respective weighted relational diagrams. Considering the same genomes $A=\{\,[\,\mathtt{1}\,\,\mathtt{2}\,\,\mathtt{3}\,\,\mathtt{4}\,\,\mathtt{5}\,]\,\}$ and $B=\{\,[\,\mathtt{6}\,\,\overline{\mathtt{7}}\,\,\overline{\mathtt{8}}\,\,\overline{\mathtt{9}}\,\,\mathtt{10}\,\,\mathtt{11}\,]\,\}$ as in Fig. 2, let $M_1$ (red) and $M_2$ (blue) be two distinct maximal matchings in ${\mathcal {S}}_{0.1}(A,B)$. We also represent the non-maximal matching $M_3$ (green) that is a subset of $M_2$. In the middle part we show diagrams $WR(A^{M_1},B^{M_1})$ and $WR(A^{M_2},B^{M_2})$, both with two $AB$-paths and two $AB$-cycles. In the lower part we show diagrams $WR(A^{M_\emptyset },B^{M_\emptyset })$, corresponding to the trivial empty matching $M_\emptyset$ and with two linear singletons (one $AA$-path and one $BB$-path), and $WR(A^{M_3},B^{M_3})$, with two $AB$-paths and two $AB$-cycles. The labeling $(\mathtt{X:Y})$ indicates that $\mathtt{Y}=s(\mathtt{X},M_i)$

Let $A^M$ and $B^M$ be the mapped family-based singular genomes for a given matching *M* of ${\mathcal {S}}_{x}(A,B)$. The *weighted relational diagram* of $A^M$ and $B^M$, denoted by $WR(A^M,B^M)$, is obtained by constructing the relational diagram of $A^M$ and $B^M$ and adding weights to the indel edges as follows. For each mapped *M*-unsaturated family $m \in {\mathcal {A}}(M) \cup {\mathcal {B}}(M)$, the indel edge $m^{\,\!h}m^{\,\!t}$ receives a weight $w(m^{\,\!h}m^{\,\!t})=\max \{ \sigma (uv) | uv \in {\mathcal {S}}_{x}(A,B) \text { and } u=s^{-1}(m,M)\}$, that is the maximum similarity among the edges incident to the marker $u=s^{-1}(m,M)$ in ${\mathcal {S}}_{x}(A,B)$. We denote by ${\widetilde{M}}= E_{\text {id}}^{A} \cup E_{\text {id}}^{B}$ the set of indel edges, here also called the *complement* of *M*. The weight of ${\widetilde{M}}$ is $w({\widetilde{M}}) = \sum _{e \in {\widetilde{M}}} w(e)$. Examples of diagrams of mapped genomes are shown in Fig. 3.

In the computation of the weighted DCJ-indel distance of mapped genomes $A^M$ and $B^M$, denoted by $wd_{DCJ}^{~id}(A^M, B^M)$, we should take into consideration the markers from exclusive families ${\mathcal {A}}(M)$ and ${\mathcal {B}}(M)$, and the weights *w*(*M*) and $w({\widetilde{M}})$. An important condition is that $wd_{DCJ}^{~id}(A^M, B^M)$ must be equal to $d_{DCJ}^{~id} (A^M, B^M)$ if $w(M)=|M|$ and $w({\widetilde{M}})=0$. We can achieve this by extending the formula for computing $wd_{DCJ}(A^M, B^M)$ as follows:

$$\begin{aligned} wd_{DCJ}^{~id}(A^M,B^M)&= wd_{DCJ}(A^M, B^M) + ~ \sum \limits _{{C\in WR(A^M,B^M)}} \lambda (C) ~ - \delta _M+ w({\widetilde{M}}) \\&= d_{DCJ} (A^M, B^M) + |M| - w(M) + ~ \sum \limits _{{C\in WR(A^M,B^M)}} \lambda (C) ~ - \delta _M+ w({\widetilde{M}}) \\&= d_{DCJ}^{~id} (A^M, B^M) + |M| - w(M) + w({\widetilde{M}}). \end{aligned}$$

Let us examine the behavior of the formula above for the examples given in Fig. 3. Matching $M_1$ is maximal and gives the distance $wd_{DCJ}^{~id}(A^{M_1},B^{M_1})=8.6$. Matching $M_2$ is also maximal and gives the distance $wd_{DCJ}^{~id}(A^{M_2},B^{M_2})=5.2$. The empty matching $M_\emptyset$ gives the distance $wd_{DCJ}^{~id}(A^{M_\emptyset },B^{M_\emptyset })=9.7$, that is the biggest. And the non-maximal matching $M_3 \subset M_2$ gives the distance $wd_{DCJ}^{~id}(A^{M_3},B^{M_3})=5.1$, that is the smallest.

Given that ${\mathbb {M}}$ is the set of all distinct matchings in ${\mathcal {S}}_{x}(A, B)$, the family-free DCJ-indel distance is defined as follows:

$$\begin{aligned} ffd_{DCJ}^{~id} (A, B, {\mathcal {S}}_{x}) = \min _{M \in {\mathbb {M}}}\{ wd_{DCJ}^{~id}(A^M,B^M) \} . \end{aligned}$$

#### Allowing matchings of any size

Other approaches that use genomic distances to disambiguate multiple connections (e.g. family-free DCJ distance [15] and DCJ-indel distance of family-based natural genomes [16]) must maximize the homology matching. The reason behind this restriction is avoiding the free lunch artifact that would otherwise let empty or almost empty matchings give smaller distances.

In contrast, here our weighting scheme prevents the free lunch and allows matchings of any size in the solution space of the family-free DCJ-indel distance. This can be explained by the fact that the adopted weights allow the family-free DCJ-indel distance to compute the exact DCJ-indel distance of family-based singular genomes [these must be properly transformed into family-free genomes together with their similarity graph by a procedure whose details can be found in Additional file 1: Appendix S1, Section (1B). The family-free DCJ-indel distance is therefore more flexible than the approaches mentioned above.

#### Complexity

Computing the family-free DCJ-indel distance is an NP-hard problem and a proof of this statement is provided in Additional file 1: Appendix S1, Section (1C).

## Family-free relational diagram

An efficient way to solve the family-free DCJ-indel distance is to develop an ILP that searches for its solution in a general graph, that represents all possible diagrams corresponding to all candidate matchings, in a similar way as the approaches given in [12, 15, 16]. Given two genomes *A* and *B* and their marker similarity graph ${\mathcal {S}}_{x}(A,B)$, the structure that integrates the properties of all possible weighted relational diagrams of mapped genomes is the *family-free relational diagram* $FFR(A,B,{\mathcal {S}}_{x})$, that has a set *V*(*A*) with a vertex for each of the two extremities of each marker of genome *A* and a set *V*(*B*) with a vertex for each of the two extremities of each marker of genome *B*.

**Fig. 4:**
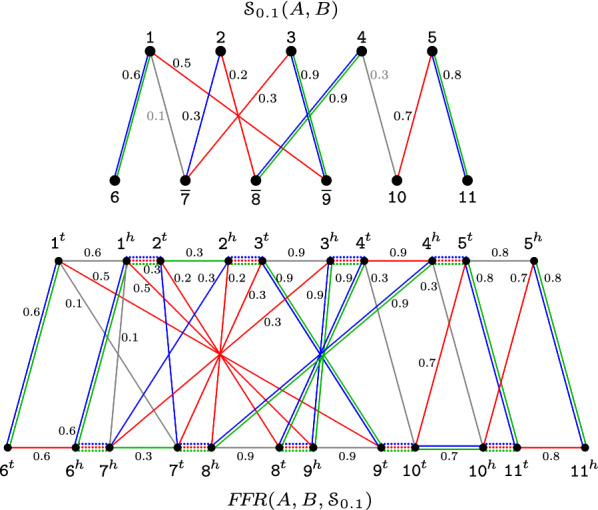
Family-free relational diagram. Given genomes $A=\{\,[\,\mathtt{1}\,\,\mathtt{2}\,\,\mathtt{3}\,\,\mathtt{4}\,\,\mathtt{5}\,]\,\}$ and $B=\{\,[\,\mathtt{6}\,\,{\mathtt{7}}\,\,{\mathtt{8}}\,\,{\mathtt{9}}\,\,\mathtt{10}\,\,\mathtt{11}\,]\,\}$, the upper part displays the marker similarity graph ${\mathcal {S}}_{0.1}(A,B)$ and the lower part displays the family-free relational diagram $FFR(A,B,{\mathcal {S}}_{0.1})$. We represent in multiple colors the edges that correspond to multiple matchings

Again, sets $E_{\text {adj}}^{A}$ and $E_{\text {adj}}^{B}$ contain adjacency edges connecting adjacent extremities of markers in *A* and in *B*. But here the set $E_{\gamma }$ contains, for each edge $ab \in {\mathcal {S}}_{x}(A,B)$, an extremity edge connecting $a^{\,\!t}$ to $b^{\,\!t}$, and an extremity edge connecting $a^{\,\!h}$ to $b^{\,\!h}$. To both edges $a^{\,\!t}b^{\,\!t}$ and $a^{\,\!h}b^{\,\!h}$, that are called *siblings*, we assign the same weight, that corresponds to the similarity of the edge *ab* in ${\mathcal {S}}_{x}(A,B)$: $w(a^{\,\!t}b^{\,\!t})=w(a^{\,\!h}b^{\,\!h})=\sigma (ab)$. Furthermore, for each marker *m* there is an indel edge connecting the vertices $m^{\,\!h}$ and $m^{\,\!t}$. The indel edge $m^{\,\!h}m^{\,\!t}$ receives a weight $w(m^{\,\!h}m^{\,\!t})=\max \{ \sigma (mv) | mv \in {\mathcal {S}}_{x}(A,B) \}$, that is, it is the maximum similarity among the edges incident to the marker *m* in ${\mathcal {S}}_{x}(A,B)$. We denote by $E_{\text {id}}^{A}$ the set of indel edges of markers in genome *A* and by $E_{\text {id}}^{B}$ the set of indel edges of markers in genome *B*. An example of a family-free relational diagram is given in Fig. 4.

### Consistent decompositions

The diagram $FFR(A,B,{\mathcal {S}}_{x})$ may contain vertices of degree larger than two. A *decomposition* of $FFR(A,B,{\mathcal {S}}_{x})$ is a collection of vertex-disjoint *components*, that can be cycles and/or paths, covering all vertices of $FFR(A,B,{\mathcal {S}}_{x})$. There can be multiple ways of selecting a decomposition, and we need to find one that allows to identify a matching of ${\mathcal {S}}_{x}(A,B)$. A set $S \subseteq E_{\gamma }$ is a *sibling-set* if it is exclusively composed of pairs of siblings and does not contain any pair of incident edges. Thus, a sibling-set *S* of $FFR(A,B,{\mathcal {S}}_{x})$ corresponds to a matching of ${\mathcal {S}}_{x}(A,B)$. In other words, there is a clear bijection between matchings of ${\mathcal {S}}_{x}(A,B)$ and sibling-sets of $FFR(A,B,{\mathcal {S}}_{x})$ and we denote by $M_S$ the matching corresponding to the sibling-set *S*.

The set of edges *D*[*S*] *induced* by a sibling-set *S* is said to be a *consistent decomposition* of $FFR(A,B,{\mathcal {S}}_{x})$ and can be obtained as follows. In the beginning, *D*[*S*] is the union of *S* with the sets of adjacency edges $E_{\text {adj}}^{A}$ and $E_{\text {adj}}^{B}$. We then need to determine the *complement* of the sibling-set *S*, denoted by ${\widetilde{S}}$, that is composed of the indel-edges of $FFR(A,B,{\mathcal {S}}_{x})$ that must be added to *D*[*S*]: for each indel edge *e*, if its two endpoints have degree one or zero in *D*[*S*], then *e* is added to both ${\widetilde{S}}$ and *D*[*S*]. (Note that ${\widetilde{S}}={\widetilde{M}}_S$, while $|S| = 2|M_S|$ and $w(S)=2w(M_S)$.) The consistent decomposition *D*[*S*] covers all vertices of $FFR(A,B,{\mathcal {S}}_{x})$ and is composed of cycles and paths, allowing us to compute the values

$$\begin{aligned} d_{DCJ}^{~id} (D[S])= & {} \frac{|S|}{2} - c_{D} - \frac{i_{D}}{2} +\sum _{C \in D[S]} \lambda (C) -\delta _{D} \, \text{ and }\\ wd_{DCJ}^{~id}(D[S])= & {} d_{DCJ}^{~id} (D[S]) + \frac{|S|}{2}- \frac{w(S)}{2} + w({\widetilde{S}}) \,, \end{aligned}$$

where $c_{D}$ and $i_{D}$ are the numbers of $AB$-cycles and $AB$-paths in *D*[*S*], respectively, and $\delta _{D}$ is the optimal deduction of recombinations of paths from *D*[*S*].

Given that ${\mathbb {S}}$ is the sets of all sibling-sets of $FFR(A,B,{\mathcal {S}}_{x})$, we compute the family-free DCJ-indel distance of *A* and *B* with the following equation:

$$\begin{aligned} ffd_{DCJ}^{~id} (A, B, {\mathcal {S}}_{x}) = \min _{S \in {\mathbb {S}}}\{wd_{DCJ}^{~id}(D[S]) \}. \end{aligned}$$

### Capping

Telomeres produce some difficulties for the decomposition of $FFR(A,B,{\mathcal {S}}_{x})$, and a known technique to overcome this problem is called *capping* [3]. It consists of modifying the diagram by adding *artificial* markers, also called *caps*, whose extremities should be properly connected to the telomeres of the linear chromosomes of *A* and *B*. Therefore, usually the capping depends on the numbers $\kappa _{A}$ and $\kappa _{B}$, that are, respectively, the total numbers of linear chromosomes in genomes *A* and *B*.

#### Family-based singular genomes

First we recall the capping of family-based singular genomes. Here the caps must circularize all linear chromosomes, so that their relational diagram is composed of cycles only, but, if the capping is optimal, the DCJ-indel distance is preserved.

An optimal capping that transforms singular linear genomes *A* and *B* into singular circular genomes can be obtained after identifying the recombination groups [6]. The DCJ-indel distance is preserved by properly linking the components of each identified recombination group into a single cycle [16]. Such a capping may require some artificial adjacencies between caps. The following result is very useful.

##### **Theorem 1**

(from [16]) *We can obtain an optimal capping of singular genomes* *A* *and* *B* *with exactly* $p_* = \max \{\kappa _{A},\kappa _{B}\}$ *caps and* $|\kappa _{A}-\kappa _{B}|$ *artificial adjacencies between caps.*

#### Capped family-free relational diagram

The diagram $FFR(A,B,{\mathcal {S}}_{x})$ is transformed into the *capped family-free relational diagram* $FFR_{\circ }(A,B,{\mathcal {S}}_{x})$ as follows. Add to $FFR(A,B,{\mathcal {S}}_{x})$ $4p_*$ new vertices, named $\circ _{A}^{1},\circ _{A}^{2},\ldots ,\circ _{A}^{2p_*}$ and $\circ _{B}^{1}, \circ _{B}^{2},\ldots ,\circ _{B}^{2p_*}$, each one representing a *cap extremity*. Connect each of the $2\kappa _{A}$ telomeres of *A* by an adjacency edge to a distinct cap extremity among $\circ _{A}^{1},\circ _{A}^{2},\ldots ,\circ _{A}^{2\kappa _{A}}$. Similarly, connect each of the $2\kappa _{B}$ telomeres of *B* by an adjacency edge to a distinct cap extremity among $\circ _{B}^{1},\circ _{B}^{2},\ldots ,\circ _{B}^{2\kappa _{B}}$. Moreover, if $\kappa _{A} < \kappa _{B}$, for $i = 2\kappa _{A}+1,2\kappa _{A}+3,\ldots ,2\kappa _{B}-1$, connect $\circ _{A}^{i}$ to $\circ _{A}^{i+1}$ by an *artificial adjacency edge*. Otherwise, if $\kappa _{B} < \kappa _{A}$, for $j = 2\kappa _{B}+1,2\kappa _{B}+3,\ldots ,2\kappa _{A}-1$, connect $\circ _{B}^{j}$ to $\circ _{B}^{j+1}$ by an artificial adjacency edge. All these new adjacency edges and artificial adjacency edges are added to $E_{\text {adj}}^{A}$ and $E_{\text {adj}}^{B}$, respectively. Finally, connect each $\circ _{A}^{i}$, $1\le i\le 2p_*$, by a *cap extremity edge* to each $\circ _{B}^{j}$, $1\le j\le 2p_*$, and denote by $E_{\circ }$ the set of cap extremity edges.

A set $P \subseteq E_{\circ }$ is a *capping-set* if it does not contain any pair of incident edges and is maximal. Since each cap extremity of *A* is connected to each cap extremity of *B*, the size of any (maximal) capping-set is $2p_*$. A *capped consistent decomposition* *Q*[*S*, *P*] of $FFR_{\circ }(A,B,{\mathcal {S}}_{x})$ is induced by a sibling-set $S \subseteq E_\gamma$ and a (maximal) capping-set $P \subseteq E_\circ$ and is composed of vertex disjoint cycles that cover all vertices of $FFR_{\circ }(A,B,{\mathcal {S}}_{x})$. An example of a capped family-free relational diagram is given in Additional file 1: Figure S1-2, Appendix S1, Section (1A).

##### **Theorem 2**

*Let* ${\mathbb {P}}_{max}$ *be the set of all distinct (maximal) capping-sets from* $FFR_{\circ }(A,B,{\mathcal {S}}_{x})$. *For each sibling-set* *S* *of* $FFR(A,B,{\mathcal {S}}_{x})$ and $FFR_{\circ }(A,B,{\mathcal {S}}_{x})$, *we have*

$$\begin{aligned} d_{DCJ}^{~id} (D[S])= & {} \min _{P \in {\mathbb {P}}_{max}}\{ d_{DCJ}^{~id} (Q[S,P])\}\,, \text{ and } \\ wd_{DCJ}^{~id}(D[S])= & {} \min _{P \in {\mathbb {P}}_{max}}\{ wd_{DCJ}^{~id}(Q[S,P])\}. \end{aligned}$$

##### *Proof*

Each capping-set corresponds to exactly $p_*$ caps. In addition, all adjacencies, including the $|\kappa _A - \kappa _B|$ artificial adjacencies between cap extremities, are part of each capped consistent decomposition. Recall that each sibling-set *S* of $FFR_{\circ }(A,B,{\mathcal {S}}_{x})$ corresponds to a matching $M_S$ of ${\mathcal {S}}_{x}(A,B)$. The set of capped consistent decompositions include all possible distinct decompositions induced by *S* together with one distinct element of ${\mathbb {P}}_{max}$. Theorem 1 states that the pair of matched genomes $A^{M_S}$ and $B^{M_S}$ can be optimally capped with $p_*$ caps and $|\kappa _A - \kappa _B|$ artificial adjacencies. Therefore, it is clear that $d_{DCJ}^{~id} (D[S]) = \min _{P \in {\mathbb {P}}_{max}}\{ d_{DCJ}^{~id} (Q[S,P])\}$. Since the capping does not change the sizes of the sibling-sets and their weights and complements, it is also clear that $wd_{DCJ}^{~id}(D[S]) = \min _{P \in {\mathbb {P}}_{max}}\{ wd_{DCJ}^{~id}(Q[S,P])\}$. $\square$

Given that ${\mathbb {S}}$ and ${\mathbb {P}}_{max}$ are, respectively, the sets of all sibling-sets and all maximal capping-sets of $FFR_{\circ }(A,B,{\mathcal {S}}_{x})$, the final version of our optimization problem is

$$\begin{aligned} ffd_{DCJ}^{~id} (A, B,{\mathcal {S}}_{x}) = \min _{S \in {\mathbb {S}}, P \in {\mathbb {P}}_{max}}\big \{wd_{DCJ}^{~id}(Q[S,P])\big \}. \end{aligned}$$

#### Alternative formula for computing the indel-potential of cycles

The capped consistent decompositions of the diagram $FFR_{\circ }(A,B,{\mathcal {S}}_{x})$ are composed exclusively of cycles, and the number of runs $\Lambda (C)$ of a cycle *C* is always in $\{0,1,2,4,6,\ldots \}$. Therefore, the formula to compute the indel-potential of a cycle *C* can be simplified to

$$\begin{aligned} \lambda (C) = {\left\{ \begin{array}{ll} \quad \;\, \Lambda (C)\,, &{} \text{ if } \Lambda (C)\in \{0,1\} \\ 1+\frac{\Lambda (C)}{2}\,, &{} \text{ if } \Lambda (C) \in \{2,4,6,\ldots \} \end{array}\right. } \end{aligned}$$

that can still be redesigned to a form that can be easier implemented in the ILP [16]. First, let a *transition* in a cycle *C* be an indel-free segment of *C* that is between a run in one genome and a run in the other genome and denote by $\aleph (C)$ the number of transitions in *C*. Observe that, if *C* is indel-free, then obviously $\aleph (C) = 0$. If *C* has a single run, then we also have $\aleph (C) = 0$. On the other hand, if *C* has at least 2 runs, then $\aleph (C)=\Lambda (C)$. The new formula is split into two parts. The first part is the function *r*(*C*), defined as $r(C)=1$ if $\Lambda (C)\ge 1$, otherwise $r(C)=0$, that simply tests whether *C* is indel-enclosing or indel-free. The second part depends on the number of transitions $\aleph (C)$, and the complete formula stands as follows [16]:

$$\begin{aligned} \lambda (C)=r(C) + \frac{\aleph (C)}{2}. \end{aligned}$$

#### New formula for computing the weighted distance

Note that the number of indel-enclosing components is $\sum _{C \in Q[S,P]} r(C) = c^{r}_{Q} + s_{Q}$, where $c^{r}_{Q}$ and $s_{Q}$ are the number of indel-enclosing $AB$-cycles and the number of circular singletons in *Q*[*S*, *P*], respectively. Furthermore, the number of indel-free $AB$-cycles of *Q*[*S*, *P*] is $c^{{\tilde{r}}}_{Q}=c_Q-c^{r}_{Q}$. We can now compute the values

2 $$\begin{aligned} d_{DCJ}^{~id} (Q[S,P])&= p_* + \frac{|S|}{2} - c_{Q} +\sum _{C \in Q[S,P]} \lambda (C)\nonumber \\&= p_* + \frac{|S|}{2} - c_{Q} +\sum _{C \in Q[S,P]} \left( r(C) + \frac{\aleph (C)}{2}\right) \nonumber \\&= p_* + \frac{|S|}{2} - c^{{\tilde{r}}}_{Q} + s_{Q} + \sum _{C \in Q[S,P]} \frac{\aleph (C)}{2}\,, \text{ and }\nonumber \\ wd_{DCJ}^{~id}(Q[S,P])&= d_{DCJ}^{~id} (Q[S,P]) + \frac{|S|}{2} - \frac{w(S)}{2} + w({\widetilde{S}}) \nonumber \\&= p_* + |S| - c^{{\tilde{r}}}_{Q} + s_{Q} + \sum _{C \in Q[S,P]} \frac{\aleph (C)}{2} - \frac{w(S)}{2}+ w({\widetilde{S}}). \end{aligned}$$

### Cutting threshold

The family-free DCJ-indel distance $ffd_{DCJ}^{~id}$ was designed to be computed with all given pairwise similarities, i.e., with the cutting threshold $x=0$, that leads to a “complete” family-free relational diagram. Such a diagram would be too large to be handled in practice, therefore, if $x=0$, we consider only the similarities that are strictly greater than 0. Nevertheless, for bigger instances the diagram with similarities close to 0 might still be too large to be solved in reasonable time. Hence, for some instances it may be necessary to do a small increase of the cutting threshold. We usually adopt a small cutting threshold up to 0.3.

## ILP formulation to compute the family-free DCJ-indel distance

Our formulation is an adaptation of the ILP for computing the DCJ-indel distance of family-based natural genomes, by Bohnenkämper et al. [16], that is itself an extension of the ILP for computing the DCJ distance of family-based balanced genomes, by Shao et al. [12]. The main differences between our approach and the approach from [16] are the underlying graphs and the objective functions. The general idea is searching for a sibling-set, that, together with a maximal capping-set, gives an optimal consistent cycle decomposition of the capped diagram $FFR_{\circ }(A,B,{\mathcal {S}}_{x}) = (V,E)$, where the set of edges comprises all disjoint sets of distinct types: $E=E_\gamma \cup E_\circ \cup E_{\text {adj}}^{A} \cup E_{\text {adj}}^{B} \cup E_{\text {id}}^{A} \cup E_{\text {id}}^{B}$. While in the ILP from [16] the search space is restricted to maximal sibling-sets, in the family-free DCJ-indel distance the search space includes all sibling-sets, of any size.

In Algorithm 1 we give the formulation for computing $ffd_{DCJ}^{~id} (A,B,{\mathcal {S}}_{x})$, distributed in three main parts. Counting indel-free cycles in the decomposition makes up the first part, depicted in constraints (C.01)–(C.06), variables and domains (D.01)–(D.03). The second part is for counting transitions, described in constraints (C.07)–(C.10), variables and domains (D.04)–(D.05). The last part describes how to count the number of circular singletons, with constraint (C.11), variable and domain (D.06). The objective function of our ILP minimizes the size of the sibling-set, with sum over variables $x_e$, the number of circular singletons, calculated by the sum over variables $s_k$, half the overall number of transitions in indel-enclosing $AB$-cycles, calculated by the sum over variables $t_e$, and the weight of all indel edges in the decomposition, given by the sum over their weights $w_ex_e$ for all $e \in E_{\text {id}}^{}$, while maximizing both the number of indel-free cycles, counted by the sum over variables $z_i$, and half of the weights of the edges in the decomposition, given by the sum over their weights $w_ex_e$ for all edges $e \in E_\gamma$. The minimization is not affected by constant $p_*$, that is included in the objective function to keep the correspondence to Eq. (2).

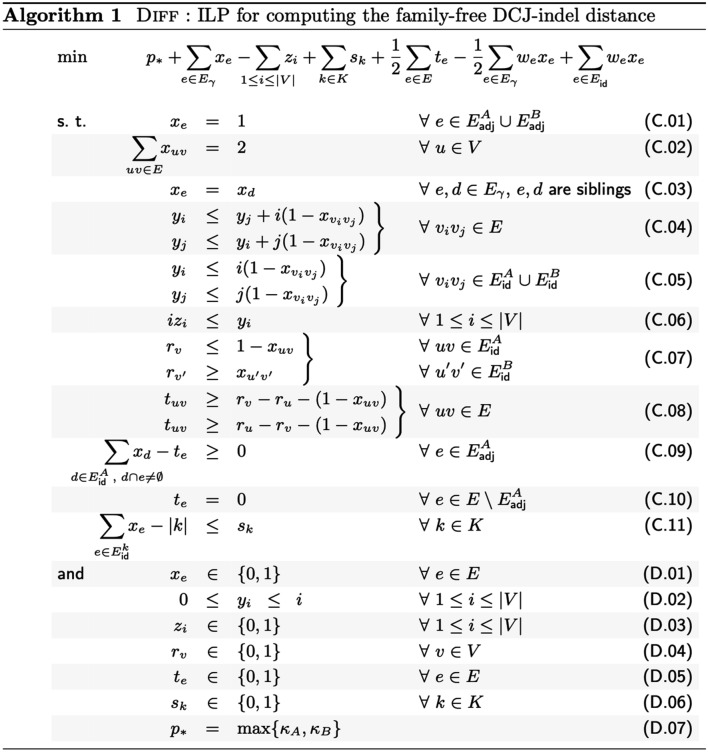



### Implementation

The ILP for computing the family-free DCJ-indel distance can be downloaded from our GitLab server at https://gitlab.ub.uni-bielefeld.de/gi/gen-diff. In the remainder of this paper it will be referred to as $DIFF$.

## Experiments

For all pairwise comparisons, we obtained gene similarities using the FFGC pipeline^2^ [19], with the following parameters: (i) 1 for the minimum number of genomes for which each gene must share some similarity in, (ii) 0.1 for the stringency threshold, (iii) 1 for the BLAST e-value, and (iv) default values for the remaining parameters.

### ILP solver and processing environment

In all our experiments we used the ILP solver CPLEX with 8 2.67GHz cores.

### Performance evaluation of $DIFF$ on simulated genomes

We generated simulated genomes using Artificial Life Simulator (ALF) [20] in order to benchmark our algorithm for computing the family-free DCJ-indel distance. We simulated and compared 190 pairs of genomes with different duplication rates, keeping all other parameters fixed (e.g. rearrangement, indel and mutation rates). The extant genomes have around 10,000 genes. We obtained gene similarities between simulated genomes using FFGC [19], as previously mentioned, and adopted a cutting threshold of $x=0.1$. This resulted in similarity graphs with up to 8400 genes with multiple connections (i.e. vertices with degree $> 1$ in ${\mathcal {S}}_{0.1}$) and with an average of 2.5 connections per gene. In addition, for each pair the genomes are about 3000 rearrangement events away from each other. The complete parameter sets used for running ALF, together with additional information on simulated genomes, can be found in Additional file 1: Appendix 2, Section (2A).

**Fig. 5:**
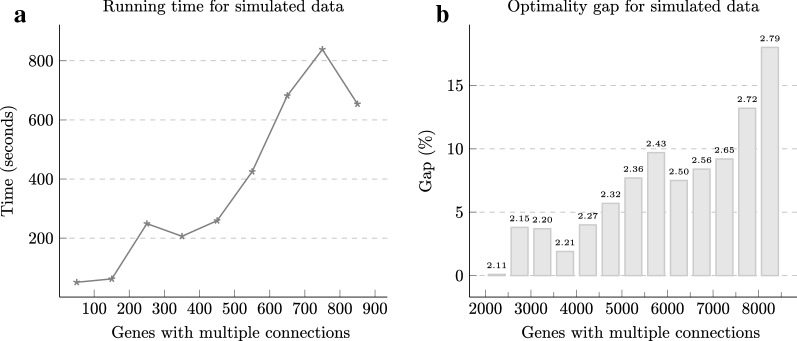
Performance of the ILP computing the family-free DCJ-indel distance of simulated genomes. The experiment results are displayed in two parts and in both of them instances are grouped by the number of genes with multiple connections (i.e. vertices with degree $> 1$ in ${\mathcal {S}}_{0.1}$): **a** shows the average running time for instances grouped in intervals of 100 and up to 900, and **b** shows the average optimality gap and the average number of connections for groups of instances that did not finish within the time limit of 1 h (in intervals of 500)

For computing the family-free DCJ-indel distances for these simulated genome pairs, we ran CPLEX with maximum CPU time of 1 h. Figure 5 summarizes the performance of $DIFF$ showing the pairwise comparisons grouped depending on the respective number of genes with multiple connections. The running times escalate quickly as the number of genes with multiple connections increase (Fig. 5a, grouped in intervals of 100), reaching the time limit after 2000 of them (Fig. 5b, grouped in intervals of 500). The optimality gap is the relative gap between the best solution found and the upper bound found by the solver, calculated by $(\frac{\text {upper bound}}{\text {best solution}} - 1) \times 100$, and appears to grow, for our simulated data, linearly in the number of genes with multiple connections (Fig. 5b).

The solution time and the optimality gap of our algorithm clearly depends less on genome sizes and more on the multiplicity of connections. In our experiments, we were able to find in 1 h optimal or near-optimal solutions for genomes with 10,000 genes and up to 4000 genes with 2.2 connections on average. Our formulation should be able to handle, for instance, the complete genomes of bacteria, fungi and insects, or even sets of chromosomes of mammal and plant genomes.

### Real data analysis

For all ILP computations described in this subsection we ran CPLEX with maximum CPU time of 3 h. [Additional tables and figures referred to here can be found in Additional file 1: Appendix S2, Section (2B)].

We evaluated the potential of our approach by doing a comparative analysis of fruit flies from the genus *Drosophila*, including the following species: *D. busckii*, *D. melanogaster*, *D. pseudoobscura*, *D. sechellia*, *D. simulans* and *D. yakuba* [21–24]. Each genome has approximately 150Mb, with about 13,000 genes distributed in 5–6 chromosomes. The sources of the genome assemblies used in our experiments are given in Additional file 1: Table S1.

The same assemblies were used by Bohnenkämper et al. [16] to evaluate the performance of their ILP that is called $DING$ and computes the related family-based DCJ-indel distance $nd_{DCJ}^{~id}$ of natural genomes (in their work, they computed OMA orthologies [25] to derive the gene families of *Drosophila* genomes, resulting in 12,735 families present in at least two genomes with 1.04 occurrences in each genome on average and at most 23 occurrences). We reproduced here the analysis done in [16] by running $DING$ in our processing environment, with the same families derived by OMA. The running time of CPLEX for each pairwise comparison was very fast, ranging from 2 to 32 s.

For our analysis with $DIFF$, pairwise gene similarities for the six *Drosophila* genomes were computed using FFGC [19], as previously described. The distribution of obtained similarities is detailed in Additional file 1: Table S2. Considering similarities that are strictly greater than $x=0$, we obtained pairwise similarity graphs with an average of 11.2 connections per gene, some of them having up to 95 connections. Since these instances were too large, we set the cutting threshold to $x=0.3$, resulting in similarity graphs with an average of 1.92 and at most 31 connections per gene. The full list including the numbers of genes with 0, 1 and multiple connections for each resulting ${\mathcal {S}}_{0.3}$ is given in Additional file 1: Table S3. All CPLEX computations of $DIFF$ on these graphs finished within the time limit, most of them in less than 10 minutes (the complete list of running times are given in Additional file 1: Table S4).

#### Assessing the quality of the results

For the three species *D. melanogaster*, *D. simulans* and *D. yakuba* we obtained reference gene families (homolog gene sets) from Flybase [26] (release FB2020_04). We classified pairs of homologous genes inferred with $DIFF$ calculations for pairwise comparisons involving these three species into four classes, listed together with their respective resulting average percentages:

(i)  *Match* (97.3%): both genes are in the same (Flybase) family;
(ii)  *New* (1.4%): both genes are not part of any family;
(iii) *Extension* (1.1%): one of the two genes is not part of any family;
(iv) *Mismatch* (0.2%): each gene is in a different family.

These results show that genes were associated with high fidelity. The complete list of homologies inferred by $DIFF$ can be found in Additional file 2.

**Fig. 6:**
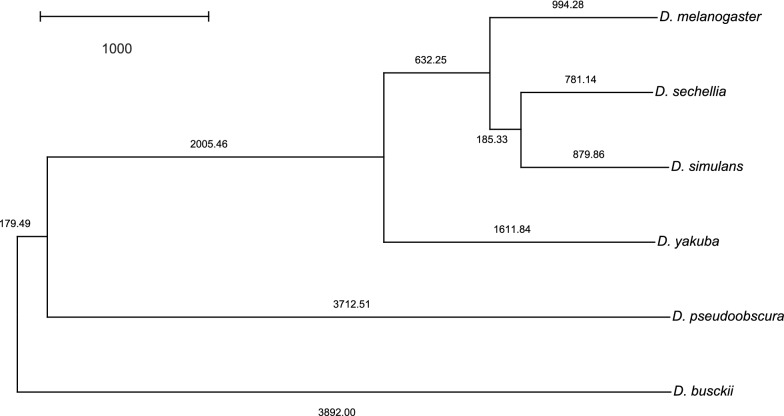
Phylogenetic tree computed based on the distances given by the family-free approach. This tree was computed by the Neighbor-Joining method [27, 28] based on distance matrices of pairwise comparisons of complete *Drosophila* genomes calculated by $DIFF$

**Fig. 7:**
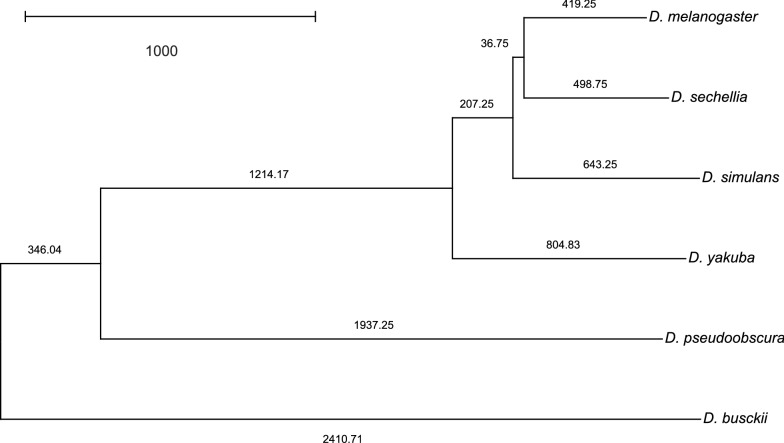
Phylogenetic tree computed based on the distances given by the family-based approach. This tree, reproduced from the results originally published in [16], was computed by the Neighbor-Joining method based on distance matrices of pairwise comparisons of complete *Drosophila* genomes calculated by $DING$. The gene families here were generated in [16] by computing OMA orthologies [25] on the same genome assemblies used in the present study

The distances computed by $DIFF$ were then used to build a phylogenetic tree using Neighbor-Joining [27, 28].^3^ The resulting tree is shown in Fig. 6 and is very similar to the reference phylogenetic tree of the six Drosophila species, generated by TimeTree [29] and shown in Additional file 1: Figure S2-1. Indeed, $DIFF$ appears to have generated a phylogenetic tree that is slightly more accurate when we compare it to the one shown in Fig. 7, obtained using Neighbor-Joining on the distances computed by $DING$ in [16]. This indicates that, besides the advantage of directly inferring homologies without pre-defined families, the flexibility of not maximizing matched genes might play an important role in obtaining better results.

#### Assessing the running times

It is not possible to fairly compare the previously mentioned running times of $DING$ and $DIFF$ because the underlying relational graphs differ in the number of connections between genes (i.e. family sizes in $DING$ versus number of edges in similarity graphs in $DIFF$). In an effort to shed some light on this matter, we devised the following experiment to balance that number for both models.

- First, we used a simple approach to convert our pairwise similarity graphs (with cutting threshold of $x=0.3$) into families: for each graph, all markers that belong to the same connected component were defined to belong to one family. All but one computations of $DING$ for these instances reached the time limit of 3 h.
- Second, we transformed each connected component in each similarity graph into a bipartite clique by adding extra edges with weight 0.3 (the same as the cutting threshold). With these extended graphs, $DIFF$ reached the time limit of 3 h for only one instance, taking 380 s on average for the remaining ones.

Note that $DING$, in spite of having a much smaller search space only composed of maximal sibling-sets, took considerably longer. This is probably due to a large number of co-optimal solutions in $nd_{DCJ}^{~id}$ that must be handled by $DING$, while in $ffd_{DCJ}^{~id}$ the co-optimality is reduced by weights, which helps $DIFF$ to converge faster: indeed, in a simulation in which the weights of all edges of the similarity graphs were set to 1, the running times of $DIFF$ were much slower than those of $DING$ for instances with the same number of multiple connections.

#### A note on the length of indel segments

As a generalization of the singular DCJ-indel model [6], the basic idea behind our approach is that runs can be merged and accumulated with DCJ operations. Note that the singular DCJ-indel model minimizes the number of indels and DCJ operations together, allowing a space of trade-off between DCJ and indel operations. Therefore it allows, up to a certain limit, co-optimal scenarios to have less DCJs and more indels, or more DCJs and less indels. This is a more elaborated and parsimonious alternative to the trivial approach of inserting or deleting exclusive markers individually. However, it raises the question of whether the indels then tend to be very long, and whether this makes biological sense. Considering that it is possible to distribute the runs so that each indel is composed of 1–2 runs, we can say that the lengths of the runs play a major role in defining the length of indel segments. In the particular analysis of *Drosophila* complete genomes, we have an average run length of 5.1, while the maximum run length is 121. We conjecture that the long runs are mostly composed of genes that are part of a contiguous segment from the beginning, and are not really accumulated by DCJ operations. In a future work we intend to have a closer look into the long runs, so that we can characterize their structures and verify this conjecture for the *Drosophila* dataset.

## Conclusions and discussion

In this work we proposed a new genomic distance, for the first time integrating DCJ and indel operations in a family-free setting. In this setting the whole analysis requires less pre-processing and no classification of the data, since it can be performed based on the pairwise similarities of markers in both genomes. Based on the positions and orientations of markers in both genomes we build the *family-free relational diagram*. We then assign weights to the edges of the diagram, according to the given pairwise similarities. A *sibling-set* of edges corresponds to a set of matched markers in both genomes. Our approach transfers weights from the edges to matched and unmatched markers, so that, again for the first time, an optimal solution does not necessarily need to maximize the number of matched markers. Instead, the search space of our approach allows solutions composed of any number of matched markers. The computation of our new family-free DCJ-indel distance is NP-hard and we provide an efficient ILP formulation to solve it.

The experiments on simulated data show that our ILP can handle not only bacterial genomes, but also complete genomes of fungi and insects, or sets of chromosomes of mammals and plants. We performed a comparison study of six fruit fly genomes, using the obtained distances to reconstruct the phylogenetic tree of the six species, obtaining accurate results. The sibling-sets inferred by our ILP in this experiment correspond to gene homologies that are 99.8% consistent with annotated gene homologies of FlyBase [26], as only 0.2% of gene matchings connected genes of different annotated families. Comparisons with the related family-based model $nd_{DCJ}^{~id}$ [16] suggest that our $ffd_{DCJ}^{~id}$ model can deliver more accurate results and can be solved faster when the inputs are of the same sizes, with the extra advantage of bypassing the pre-identification of gene families. This study is a first validation of the quality of our method and a more rigorous evaluation will be performed in future works, including, as previously mentioned, the investigation of the reasons behind insertions and deletions of long segments in the *Drosophila* dataset.

## Supplementary information

**Additional file 1.** Supplementary material on the model and on the experiments, including supplementary figures and tables.

**Additional file 2.** Lists of inferred gene matchings for each pairwise comparison of *Drosophila* genomes and homolog gene sets (gene families) for *D. melanogaster,* *D. simulans *and *D. yakuba* defined by FlyBase (release FB2020 04) with gene identifiers converted to NCBI identifiers.

## Footnotes

1 Exclusive families are not restricted to be singular: an exclusive family that occur multiple times in a genome can be trivially split into singular families.

2 https://bibiserv.cebitec.uni-bielefeld.de/ffgc.

3 The output of this algorithm is an unrooted tree, and we assumed the most distant species *D. busckii* as the outgroup for rooting the tree.
