# Supplementary material for: Natural family-free genomic distance
Source: Algorithms Mol Biol. 2021 May 10;16:4. doi: 10.1186/s13015-021-00183-8 (PMC8111734; doi:10.1186/s13015-021-00183-8)
Supplement: Supplementary file 1 — Additional file 1. Supplementary material on the model and on the experiments, including supplementary figures and tables. [file 13015_2021_183_MOESM1_ESM.pdf]

Additional File 1 - Online supplemental material of: Natural family-free genomic distance

Diego P. Rubert[1], Fábio V. Martinez[1] and Marília D. V. Braga[2]

Appendix S1: Supplementary material on the model

(1A) Supplementary figures

Figure S1-1 shows a BB-path with 4 runs, and how its indel-potential can be achieved.

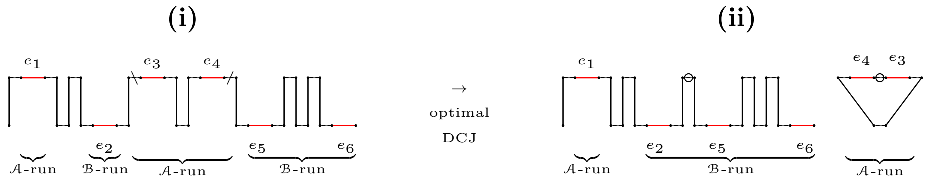

Figure S1-1: **Runs and indel-potential of a path in the relational diagram.** (i) A BB-path with 4 runs. (ii) After an optimal DCJ that creates a new cycle, one $\mathcal{A}$-run is accumulated (between edges e_4 and e_3 there is only an adjacency edge) and two $\mathcal{B}$-runs are merged (e_2 is in the same run with e_5 and e_6). Indeed the indel-potential of the original BB-path is three.

Figure S1-2 shows an example of a capped family-free relational diagram.

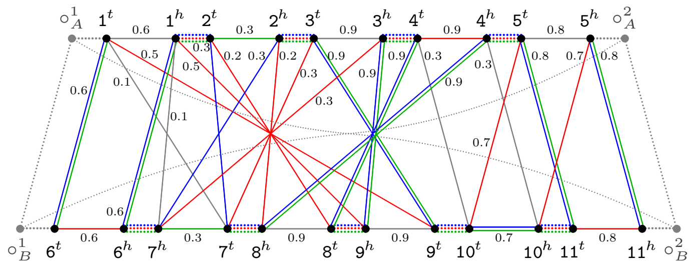

Figure S1-2: **Capped family-free relational diagram.** The capped version of the family-free relational diagram from Figure 4.

[1]Faculdade de Computação, Universidade Federal de Mato Grosso do Sul, Brazil
[2]Faculty of Technology and Center for Biotechnology (CeBiTec), Bielefeld University, Germany

(1B) Converting family-based to family-free genomes

We can transform family-based genomes into family-free genomes together with their similarity graph with the following procedure.

Definition 1 *Given family-based genomes $\breve{A}$ and $\breve{B}$, we derive family-free genomes A and B and their similarity graph $\mathcal{S}_1(A,B)$, as follows:*

1. *In the beginning A and B are just copies from $\breve{A}$ and $\breve{B}$.*
2. *Then, given that $\breve{\mathcal{F}}_\star$ is the set of common families between $\breve{A}$ and $\breve{B}$, each occurrence of a family $m \in \breve{\mathcal{F}}_\star$ is renamed to $m_\mathtt{A}$ in genome A and to $m_\mathtt{B}$ in genome B and the similarity between each $m_\mathtt{A}$ and each $m_\mathtt{B}$ is set to 1. (Note: additionally, for each family f that occurs more than once in one of the two genomes, we can differentiate the occurrences of f by assigning an arbitrary distinct index to each occurrence.)*
3. *The similarity between any pair of markers whose families in the original genomes $\breve{A}$ and $\breve{B}$ are distinct is set to 0.*

Note that, for any $0 < x \leq 1$, we have $\mathcal{S}_x(A,B) = \mathcal{S}_1(A,B)$. Examples (considering that original genomes $\breve{A}$ and $\breve{B}$ are singular) are shown in Figure S1-3.

Now let $\mathcal{G}_\star$ be the multiset of common markers of $\breve{A}$ and $\breve{B}$, given by the intersection of their multisets of markers: $\mathcal{G}_\star = \mathcal{G}(\breve{A}) \cap \mathcal{G}(\breve{A})$. Furthermore, denote $n = |\breve{\mathcal{G}}_\star|$ and let $M_n, M_{n-1}, M_{n-2}, \ldots, M_2, M_1, M_0$ be a sequence of matchings in $\mathcal{S}_1(A,B)$ such that, for any $1 \leq k \leq n$, M_{k-1} is obtained by removing one edge from M_k. The effects of such an edge removal are stated in the following propositions.

Proposition 1 *The relation between the DCJ distances of singular genomes given by matchings M_{k-1} and M_k in $\mathcal{S}_1(A,B)$ is*

$$\mathrm{d}_{\mathrm{DCJ}}(A^{M_{k-1}}, B^{M_{k-1}}) \geq \mathrm{d}_{\mathrm{DCJ}}(A^{M_k}, B^{M_k}) - 2\,.$$

Proof: The number of common markers change following $|M_{k-1}| = |M_k| - 1 = k - 1$.

The replacement of the pair of siblings (extremity edges) that connected the extremities of the removed marker by two indel-edges in the best case does not change the number of components of the graph.

Therefore, the DCJ part can decrease by 1 or by 2, the latter only if a pair of *AA*- + *BB*-path is transformed into a pair of *AB*-paths. □

Proposition 2 *The relation between the overall indel-potentials of the relational graphs R_{k-1} and R_k, that correspond respectively to singular genomes given by matchings M_{k-1} and M_k in $\mathcal{S}_1(A,B)$ is*

$$\sum_{C \in R_{k-1}} \lambda(C) \geq \sum_{C \in R_k} \lambda(C) \quad - \quad 2\,.$$

Proof: The new indel-edges added to the diagram by removing one edge from the matching can merge two pairs of runs and, consquently, can decrease the overall-indel potential in $R(A^{M_{k-1}}, B^{M_{k-1}})$ by at most 2. □

Proposition 3 *The relation between the DCJ-indel distances of singular genomes given by matchings M_{k-1} and M_k in $\mathcal{S}_1(A,B)$ is*

$$\mathrm{d}^{\mathrm{ID}}_{\mathrm{DCJ}}(A^{M_{k-1}}, B^{M_{k-1}}) \geq \mathrm{d}^{\mathrm{ID}}_{\mathrm{DCJ}}(A^{M_k}, B^{M_k}) - 2\,.$$

Proof: Propositions 1 and 2 together suggest that $\mathrm{d}^{\mathrm{ID}}_{\mathrm{DCJ}}(A^{M_{k-1}}, B^{M_{k-1}}) \geq \mathrm{d}^{\mathrm{ID}}_{\mathrm{DCJ}}(A^{M_k}, B^{M_k}) - 4$.

However, these Propositions do not take into consideration the deducting recombinations that can decrease the overall DCJ-indel distance. Indeed, after having a closer look on how these parts interfere with the possible deducting recombinations, we can show that $\mathrm{d}^{\mathrm{ID}}_{\mathrm{DCJ}}(A^{M_{k-1}}, B^{M_{k-1}}) \geq \mathrm{d}^{\mathrm{ID}}_{\mathrm{DCJ}}(A^{M_k}, B^{M_k}) - 2$.

The possible cases are the following:

1 The extremity-edges removed from M_k were in the same component c of the diagram. In this case the two new indel-edges induced by M_{k-1} will close c in a shorter component, with $d_{\text{DCJ}}(A^{M_{k-1}}, B^{M_{k-1}}) = d_{\text{DCJ}}(A^{M_k}, B^{M_k}) - 1$. The new indel-edges can reduce the number of runs in c by at most 2, therefore $\sum_{C \in R_{k-1}} \lambda(C) \geq \sum_{C \in R_k} \lambda(C) - 1$. A new deducting recombination decreasing the overall distance by $i \in \{1, 2\}$ can only appear here if the overall indel-potential increases by i.

2 The extremity-edges removed from M_k were in two distinct components, at least one of the two being a cycle. In this case the two new indel-edges induced by M_{k-1} will merge the two components together, but with overall less extremity edges, with $d_{\text{DCJ}}(A^{M_{k-1}}, B^{M_{k-1}}) = d_{\text{DCJ}}(A^{M_k}, B^{M_k})$. The new indel-edges can reduce the overall number of runs by at most 2, therefore $\sum_{C \in R_{k-1}} \lambda(C) \geq \sum_{C \in R_k} \lambda(C) - 2$. A new deducting recombination decreasing the overall distance by $i \in \{1, 2\}$ can only appear here if the overall indel-potential increases by i.

3 The extremity-edges removed from M_k were in two distinct paths p_1 and p_2 and the new indel-edges recombine them into p'_1 and p'_2. The new indel-edges can reduce the overall number of runs by at most 2, therefore $\sum_{C \in R_{k-1}} \lambda(C) \geq \sum_{C \in R_k} \lambda(C) - 2$.

   (a) If p_1 is an *AA*-path and p_2 is a *BB*-path, the new paths p'_1 and p'_2 are *AB*-paths, with $d_{\text{DCJ}}(A^{M_{k-1}}, B^{M_{k-1}}) = d_{\text{DCJ}}(A^{M_k}, B^{M_k}) - 2$. The overall indel-potential here can decrease by at most $i \in \{1, 2\}$. But if it decreases by i, this means that the diagram of M_k had a deducting recombination that reduced its overall DCJ-indel distance by i and was eliminated by the edge removal.

   (b) If p_1 and p_2 are *AB*-paths and p'_1 is an *AA*-path, while p'_2 is a *BB*-path, we have $d_{\text{DCJ}}(A^{M_{k-1}}, B^{M_{k-1}}) = d_{\text{DCJ}}(A^{M_k}, B^{M_k})$. The overall indel-potential can decrease by at most 2. A new deducting recombination decreasing the overall distance by $i \in \{1, 2\}$ can only appear here if the overall indel-potential increases by i.

   (c) If p_1 and p_2 are *AB*-paths and the new paths p'_1 and p'_2 are also *AB*-paths, with $d_{\text{DCJ}}(A^{M_{k-1}}, B^{M_{k-1}}) = d_{\text{DCJ}}(A^{M_k}, B^{M_k}) - 1$. The overall indel-potential here can decrease by at most 2. But if it decreases by 2, this means that the diagram of M_k had a deducting recombination that reduced its overall DCJ-indel distance by 1 and was eliminated by the edge removal.

   (d) If p_1 and p_2 are both *AA*-paths (respect. *BB*-paths), then the new paths p'_1 and p'_2 are also *AA*-paths (respect. *BB*-paths), with $d_{\text{DCJ}}(A^{M_{k-1}}, B^{M_{k-1}}) = d_{\text{DCJ}}(A^{M_k}, B^{M_k}) - 1$. The overall indel-potential here can decrease by at most 2. But if it decreases by 2, this means that the diagram of M_k had a deducting recombination that reduced its overall DCJ-indel distance by 1 and was eliminated by the edge removal.

□

Proposition 4 *The relation between the weights of the complements of matchings M_{k-1} and M_k in $\mathcal{S}_1(A, B)$ is*

$$w(\widetilde{M}_{k-1}) = w(\widetilde{M}_k) + 2\,.$$

Proof: All edges of matching M_k have weight 1. If one edge is removed from the matching, two markers, each of weight 1, are added to its complement. □

Propositions 3 and 4 guarantee that, when we decrease the size of a matching, the weighted DCJ-indel distance cannot decrease:

Corollary 1 *The relation between the weighted DCJ-indel distances of singular genomes given by matchings M_{k-1} and M_k in $\mathcal{S}_1(A, B)$ is*

$$\text{wd}_{\text{DCJ}}^{\text{ID}}(A^{M_k}, B^{M_k}) \leq \text{wd}_{\text{DCJ}}^{\text{ID}}(A^{M_{k-1}}, B^{M_{k-1}})\,,$$

implying that

$$\begin{aligned}
\text{wd}_{\text{DCJ}}^{\text{ID}}(A^{M_n}, B^{M_n}) &\leq \text{wd}_{\text{DCJ}}^{\text{ID}}(A^{M_{n-1}}, B^{M_{n-1}}) \\
&\leq \ldots \\
&\leq \text{wd}_{\text{DCJ}}^{\text{ID}}(A^{M_1}, B^{M_1}) \\
&\leq \text{wd}_{\text{DCJ}}^{\text{ID}}(A^{M_0}, B^{M_0})\,.
\end{aligned}$$

Corollary 1 states that the maximal matching M_n gives the smallest $\mathrm{wd}_{\mathrm{DCJ}}^{\mathrm{ID}}$ for each sequence of matchings $M_n, M_{n-1}, \ldots, M_1, M_0$, (where M_{k-1} is obtained by removing one edge from M_k), but allowing smaller matchings to give an equally small $\mathrm{wd}_{\mathrm{DCJ}}^{\mathrm{ID}}$.

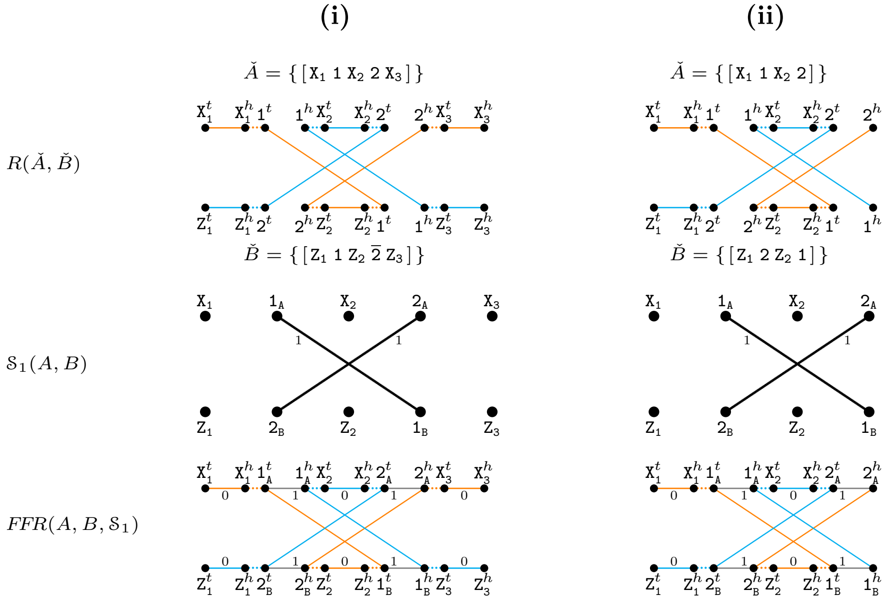

Figure S1-3: **Deriving family-free genomes and similarity graphs from two pairs of family-based singular genomes.** (i) For original singular genomes $\breve{A} = \{[\mathtt{X}_1\, 1\, \mathtt{X}_2\, 2\, \mathtt{X}_3]\}$ and $\breve{B} = \{[\mathtt{Z}_1\, 2\, \mathtt{Z}_2\, 1\, \mathtt{Z}_3]\}$, the distance $\mathrm{d}_{\mathrm{DCJ}}^{\mathrm{ID}}(\breve{A}, \breve{B}) = 6$ achieves the diameter. The derived family-free genomes are $A = \{[\mathtt{X}_1\, 1_{\mathtt{A}}\, \mathtt{X}_2\, 2_{\mathtt{A}}\, \mathtt{X}_3]\}$ and $B = \{[\mathtt{Z}_1\, 2_{\mathtt{B}}\, \mathtt{Z}_2\, 1_{\mathtt{B}}\, \mathtt{Z}_3]\}$. Consider in $\mathcal{S}_1(A, B)$ the maximal matching $M_2 = \{1_{\mathtt{A}}1_{\mathtt{B}}, 2_{\mathtt{A}}2_{\mathtt{B}}\}$ and the alternative matchings $M_1 = \{1_{\mathtt{A}}1_{\mathtt{B}}\}$ and $M_0 = \emptyset$. Observe that $\mathrm{d}_{\mathrm{DCJ}}^{\mathrm{ID}}(\breve{A}, \breve{B}) = \mathrm{wd}_{\mathrm{DCJ}}^{\mathrm{ID}}(A^{M_2}, B^{M_2}) = \mathrm{wd}_{\mathrm{DCJ}}^{\mathrm{ID}}(A^{M_1}, B^{M_1}) = \mathrm{wd}_{\mathrm{DCJ}}^{\mathrm{ID}}(A^{M_0}, B^{M_0}) = 6$. Indeed, here the distance is always 6, independently of the matching. (ii) For original singular genomes $\breve{A} = \{[\mathtt{X}_1\, 1\, \mathtt{X}_2\, 2]\}$ and $\breve{B} = \{[\mathtt{Z}_1\, 2\, \mathtt{Z}_2\, 1]\}$, the distance is $\mathrm{d}_{\mathrm{DCJ}}^{\mathrm{ID}}(\breve{A}, \breve{B}) = 4$. The derived family-free genomes are $A = \{[\mathtt{X}_1\, 1_{\mathtt{A}}\, \mathtt{X}_2\, 2_{\mathtt{A}}]\}$ and $B = \{[\mathtt{Z}_1\, 2_{\mathtt{B}}\, \mathtt{Z}_2\, 1_{\mathtt{B}}]\}$. Consider in $\mathcal{S}_1(A, B)$ the maximal matching $M_2 = \{1_{\mathtt{A}}1_{\mathtt{B}}, 2_{\mathtt{A}}2_{\mathtt{B}}\}$ and the alternative matchings $M_1 = \{1_{\mathtt{A}}1_{\mathtt{B}}\}$ and $M_0 = \emptyset$. Here $\mathrm{wd}_{\mathrm{DCJ}}^{\mathrm{ID}}(A^{M_2}, B^{M_2}) = \mathrm{d}_{\mathrm{DCJ}}^{\mathrm{ID}}(\breve{A}, \breve{B}) = 4$, but $\mathrm{wd}_{\mathrm{DCJ}}^{\mathrm{ID}}(A^{M_1}, B^{M_1}) = 5$ and $\mathrm{wd}_{\mathrm{DCJ}}^{\mathrm{ID}}(A^{M_0}, B^{M_0}) = 2 + 4 = 6$.

Computing the DCJ-indel distance of singular genomes via FF DCJ-indel distance

Assume that the original genomes $\breve{A}$ and $\breve{B}$ are singular. Here there is a unique maximal matching M_n in $\mathcal{S}_1(A, B)$. It is clear that $w(\widetilde{M_n}) = 0$, therefore $\mathrm{ffd}_{\mathrm{DCJ}}^{\mathrm{ID}}(A, B, \mathcal{S}_1) = \mathrm{wd}_{\mathrm{DCJ}}^{\mathrm{ID}}(A^{M_n}, B^{M_n}) = \mathrm{d}_{\mathrm{DCJ}}^{\mathrm{ID}}(\breve{A}, \breve{B})$.

In order to figure out the existence of co-optimal smaller matchings, let us examine the difference between $\mathrm{wd}_{\mathrm{DCJ}}^{\mathrm{ID}}(A^{M_n}, B^{M_n})$ and $\mathrm{wd}_{\mathrm{DCJ}}^{\mathrm{ID}}(A^{M_0}, B^{M_0})$ as $\mathrm{d}_{\mathrm{DCJ}}^{\mathrm{ID}}(\breve{A}, \breve{B})$ varies from its minimum to its maximum value. Let us first compute the weighted DCJ-indel distance of the empty matching:

Proposition 5 *If genomes $\breve{A}$ and $\breve{B}$ are singular, the empty matching M_0 in $\mathcal{S}_1(A, B)$ gives the following weighted DCJ-indel distance:*

$$\mathrm{wd}_{\mathrm{DCJ}}^{\mathrm{ID}}(A^{M_0}, B^{M_0}) = \chi_{\breve{A}} + \chi_{\breve{B}} + 2|\breve{\mathcal{G}}_\star|\,,$$

where $\chi_{\breve{A}}$ is the number of chromosomes in genomes A and $\breve{A}$ and $\chi_{\breve{B}}$ is the number of chromosomes in genomes B and $\breve{B}$.

Proof: Each chromosome of $\breve{A}$ and of $\breve{B}$ corresponds to an indel and the weight of the complement is $w(\widetilde{M_0}) = 2|\breve{\mathcal{G}}_\star|$. □

The DCJ-indel diameter of the family-based singular genomes $\breve{A}$ and $\breve{B}$, that is the maximum possible value of their DCJ-indel distance, is denoted by $\mathrm{D}_{\mathrm{DCJ}}^{\mathrm{ID}}(\breve{A},\breve{B})$ and can be computed with the formula established in [18]:

$$\mathrm{d}_{\mathrm{DCJ}}^{\mathrm{ID}}(\breve{A},\breve{B}) \leq \mathrm{D}_{\mathrm{DCJ}}^{\mathrm{ID}}(\breve{A},\breve{B}) = 2|\breve{\mathcal{G}}_\star| + s + \kappa_{\breve{A}} + \kappa_{\breve{B}}\,,$$

where s is the number of circular singletons in $R(A,B)$ and $\kappa_{\breve{A}}$ and $\kappa_{\breve{B}}$ are, respectively, the number of linear chromosomes in $\breve{A}$ and in $\breve{B}$. Note that each singleton is a circular chromosome, therefore $s + \kappa_{\breve{A}} + \kappa_{\breve{B}} \leq \chi_{\breve{A}} + \chi_{\breve{B}}$.

- If the distance $\mathrm{d}_{\mathrm{DCJ}}^{\mathrm{ID}}(\breve{A},\breve{B})$ tends to be big, many matchings tend to be co-optimal. In the most extreme case, when $\mathrm{d}_{\mathrm{DCJ}}^{\mathrm{ID}}(\breve{A},\breve{B}) = \mathrm{D}_{\mathrm{DCJ}}^{\mathrm{ID}}(\breve{A},\breve{B})$ and there are no circular chromosomes that are not singletons in $\breve{A}$ and in $\breve{B}$, all possible matchings give the same optimal weighted DCJ-indel distance (see Figure S1-3 (i)).
- If the distance $\mathrm{d}_{\mathrm{DCJ}}^{\mathrm{ID}}(\breve{A},\breve{B})$ tends to be small we have

$$\begin{aligned}\mathrm{d}_{\mathrm{DCJ}}^{\mathrm{ID}}(\breve{A},\breve{B}) = \mathrm{wd}_{\mathrm{DCJ}}^{\mathrm{ID}}(A^{M_n},B^{M_n}) \ll \mathrm{D}_{\mathrm{DCJ}}^{\mathrm{ID}}(\breve{A},\breve{B}) \\ \leq \mathrm{wd}_{\mathrm{DCJ}}^{\mathrm{ID}}(A^{M_0},B^{M_0}) = \chi_{\breve{A}} + \chi_{\breve{B}} + 2|\breve{\mathcal{G}}_\star|\,.\end{aligned}$$

  In this case it can be expected that the maximum matching is the unique optimal solution (see Figure S1-3 (ii)).

(1C) Computational complexity of the family-free DCJ-indel distance

In the family-based setting, if two genomes contain the same number of occurrences of each marker, they are said to be *balanced*. Notice that there are no exclusive markers in this case. The problem of computing the DCJ distance of balanced genomes is mentioned to be NP-hard [12] without a proof, though. Here we provide a simple and straightforward idea for a reduction from the DCJ double distance problem, which is shown NP-hard by Tannier *et al.* (BMC Bioinformatics, 10 (120), 2009), proving that the problem of computing the DCJ distance of balanced genomes is NP-hard.

NP-hardness of the family-based DCJ distance of balanced genomes

First, we need some well-established definitions. A *duplicated marker* m encompasses two copies of homologous oriented streches of DNA and it is identified by two copies of each extremity of m, such as m_1^t, m_1^h and m_2^t, m_2^h. An *all-duplicates genome* $\mathbb{A}$ is a set of chromosomes on a set of duplicated markers of the set of markers $\mathcal{A}$. For a given genome A on a set of markers $\mathcal{A}$, a *doubled genome* $A \oplus A$ is an all-duplicates genome on the set of duplicated markers from $\mathcal{A}$ such that if $x^e y^f$ is an adjacency of A, with $e, f \in \{t, h\}$, then either $x_1^e y_1^f$ and $x_2^e y_2^f$ or $x_1^e y_2^f$ and $x_2^e y_1^f$ are adjacencies in $A \oplus A$. In the *double distance* problem, we are given an all-duplicates genome $\mathbb{A}$ and a singular genome A, and we want to compute the distance between $\mathbb{A}$ and A. Tannier *et al.* (BMC Bioinformatics, 10 (120), 2009) showed that the DCJ double distance problem is NP-hard for multichromosomal mixed or circular genomes.

From these definitions, we can show an easy idea of proof that the DCJ distance of balanced genomes is NP-hard. Since the assignment of indices “1” or “2” to the two copies of a duplicated marker in an all-duplicates genome $\mathbb{A}$ is arbitrary, the double distance problem is equivalent to finding such an assignment that minimizes the distance between $\mathbb{A}$ and a doubled genome $A \oplus A$. Thus, the DCJ double distance problem is a particular case of the DCJ distance of balanced genomes, where each marker has exactly two copies in each genome. Therefore, the result follows.

Theorem 3 *Computing the family-based DCJ distance of balanced genomes is NP-hard.*

NP-hardness of the family-free DCJ-indel distance

Now we use the problem of computing the family-based DCJ distance of balanced genomes in a straightforward reduction to show that computing the family-free DCJ-indel distance is an NP-hard problem.

Theorem 4 *For given genomes A and B and a marker similarity graph $\mathcal{S}_x(A,B)$ for any $0 \leq x \leq 1$, computing the weighted family-free DCJ-indel distance $\mathrm{ffd}_{\mathrm{DCJ}}^{\mathrm{ID}}(A,B,\mathcal{S}_x)$ is NP-hard.*

Proof: The first step of the reduction is deriving, from family-based balanced genomes $\check{A}$ and $\check{B}$, family-free genomes A and B and their similarity graph $\mathcal{S}_1(A,B)$, following the procedure described in Definition 1.

Recall that $\mathcal{G}_\star = \mathcal{G}(\check{A}) \cap \mathcal{G}(\check{B})$ and let $n = |\mathcal{G}_\star|$. Now let $M_n, M_{n-1}, \ldots, M_1, M_0$ be a sequence of matchings in $\mathcal{S}_1(A,B)$ such that, for any $1 \leq k \leq n$, $|M_k| = k$ and M_{k-1} is obtained by removing one edge from M_k. Corollary 1 guarantees that:

$$\begin{aligned}
\mathrm{wd}_{\mathrm{DCJ}}^{\mathrm{ID}}(A^{M_n}, B^{M_n}) &\leq \mathrm{wd}_{\mathrm{DCJ}}^{\mathrm{ID}}(A^{M_{n-1}}, B^{M_{n-1}}) \\
&\leq \ldots \\
&\leq \mathrm{wd}_{\mathrm{DCJ}}^{\mathrm{ID}}(A^{M_1}, B^{M_1}) \\
&\leq \mathrm{wd}_{\mathrm{DCJ}}^{\mathrm{ID}}(A^{M_0}, B^{M_0})\,.
\end{aligned}$$

All maximal matchings in $\mathcal{S}_1(A,B)$ have cardinality n. A consequence of the result above is that there is at least one maximal matching in $\mathcal{S}_1(A,B)$ whose weighted DCJ-indel distance corresponds to $\mathrm{ffd}_{\mathrm{DCJ}}^{\mathrm{ID}}(A,B,\mathcal{S}_1)$. Since all maximal matchings give mapped canonical genomes, the weights of their complements are equal to 0, and $\mathrm{ffd}_{\mathrm{DCJ}}^{\mathrm{ID}}(A,B,\mathcal{S}_1) = \mathrm{d}_{\mathrm{DCJ}}^{\mathrm{ID}}(\check{A},\check{B}) = \mathrm{d}_{\mathrm{DCJ}}(\check{A},\check{B})$.

However, Corollary 1 also allows smaller matchings to give co-optimal solutions, and here we need to guarantee that only maximal matchings may give co-optimal solutions.

Let us then have a closer look at the effect of removing one edge from a maximal matching M_n to obtain a matching M_{n-1}:

- The number of indels clearly increase from 0 to 2: mapped genomes A^{M_n} and B^{M_n} are canonical, therefore, no indel operation can be performed on these genomes.
- The DCJ part of the formula decreases by at most 2 (Proposition 1).
- The weights of the complements are $w(\widetilde{M_n}) = 0$ and $w(\widetilde{M_{n-1}}) = 2$.

Consequently, it is clear that

$$\mathrm{wd}_{\mathrm{DCJ}}^{\mathrm{ID}}(A^{M_n}, B^{M_n}) < \mathrm{wd}_{\mathrm{DCJ}}^{\mathrm{ID}}(A^{M_{n-1}}, B^{M_{n-1}}) - 1\,.$$

□

Appendix S2:
Supplementary material on the experiments

(2A) Generation of simulated data

Here we describe the process and the parameters used in Artificial Life Simulator (ALF) [20] for generating our simulated data. Each one of the 190 instances generated consists of a pair of simulated genomes. We used the default values for parameters not mentioned. PAM units were used as time scale for simulation, starting with a randomly generated root genome with 10,000 genes, whose lengths where drawn from a Gamma distribution with $k = 2.4019$ and $\theta = 133.8063$ (minimum length 100). We used a custom evolutionary tree defining an speciation event after 25 time units, resulting in two leaf species, which evolved for additional 25 time units. The WAG substitution model was used together with Zipfian indels in DNA sequences with rate 0.0002 (maximum length 50). Such rate varies among sites according to a Gamma distribution with shape 1 and 10 classes. In addition, we set the rate of invariable sites to 0.001. Inversions and translocations of up to 30 genes were allowed at a rate of 0.0025. Finally, for generating instances comprising genes with multiple connections at various rates, we varied the gene duplication and the gene loss rates between 1×10^{-5} and 2×10^{-3}.

(2B) Analysis of Drosophila genomes

We downloaded the genomes of six species of *Drosophila* [21-24] from NCBI. In our experiments we used the assemblies listed in Table S1, with their respective gene annotations. A reference tree of these six species is displayed in Figure S2-1.

Table S1: **List of *Drosophila* genomes used in our experiments.** All six assemblies together with their respective gene annotations were downloaded from NCBI (https://www.ncbi.nlm.nih.gov)

**Species**	**NCBI Assembly**
Drosophila busckii	ASM1175060v1
Drosophila melanogaster	Release 6 plus ISO1 MT
Drosophila pseudoobscura	UCI_Dpse_MV25
Drosophila sechellia	ASM438219v1
Drosophila simulans	ASM75419v2
Drosophila yakuba	dyak_caf1

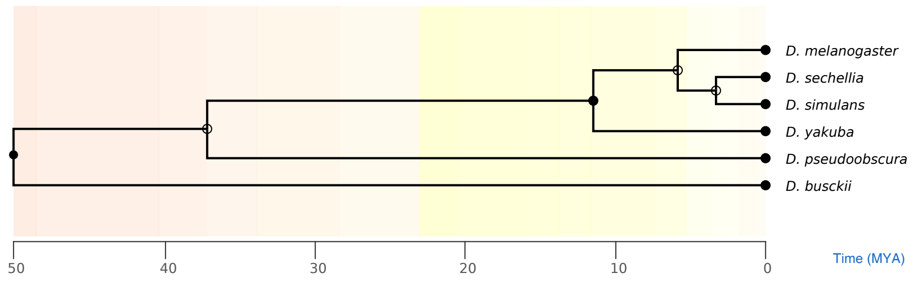

Figure S2-1: **Reference phylogenetic tree of six *Drosophila* species.** This tree was generated by TimeTree [29], a public knowledge-base for information on the tree-of-life and its evolutionary timescale.

Each genome has approximately 150Mb, with about 15,000 genes distributed in 5–6 chromosomes. Unplaced scaffolds were discarded, decreasing the number of genes, from $\sim$ 15,000 to $\sim$ 13,000.

As already mentioned, we obtained pairwise similarities between genes of *Drosophila* genomes using the FFGC pipeline [19] (`https://bibiserv.cebitec.uni-bielefeld.de/ffgc`) with the following parameters: (i) 1 for the minimum number of genomes for which each gene must share some similarity in, (ii) 0.1 for the stringency threshold, (iii) 1 for the BLAST e-value, and (iv) default values for the remaining parameters.

In the following, in-depth information is provided on the results for experiments using complete genomes of the listed *Drosophila* species. Table S2 outlines the number of gene pairs in each similarity range for each pair of genomes. Table S3 shows, considering only pairwise similarities greater or equal to 0.3, the number of genes with no connection (which induce trivial selections of indel edges in the relational diagram), the number of genes with exactly one connection and the number of genes with multiple connections (which pose a significant challenge to the solver). The computed distances and elapsed time (or gap in % when the solver reaches the time limit) in the pairwise comparisons with cutting threshold 0.3 are shown in Table S4. The solver was set to stop after finding a solution with optimality gap smaller than 0.5% or after 3 hours. The complete list of inferred homologies for all pairwise comparisons is available in Additional file 2.

Table S2: **Gene similarities in pairwise comparisons of complete genomes.** Distribution of similarities between genes (and percentage) in pairwise comparisons of complete genomes.

species	similarity	*pseudoobscura*	*sechellia*	*simulans*	*yakuba*	*busckii*
	(0.0-0.2)	53648 (60.09%)	33409 (48.69%)	34803 (49.15%)	38143 (51.71%)	53733 (65.42%)
	[0.2-0.4)	19034 (21.32%)	17822 (25.97%)	18566 (26.22%)	18748 (25.42%)	16129 (19.64%)
melanogaster	[0.4-0.6)	6036 (6.76%)	3896 (5.68%)	4019 (5.68%)	4195 (5.69%)	5207 (6.34%)
	[0.6-0.8)	4993 (5.59%)	1826 (2.66%)	1909 (2.70%)	3010 (4.08%)	4300 (5.23%)
	[0.8-1.0]	5570 (6.24%)	11666 (17.00%)	11513 (16.26%)	9663 (13.10%)	2772 (3.37%)
		89281 (100%)	68619 (100%)	70810 (100%)	73759 (100%)	82141 (100%)
	(0.0-0.2)		53777 (62.13%)	54221 (61.83%)	54147 (61.96%)	54104 (65.78%)
	[0.2-0.4)		18169 (20.99%)	18724 (21.35%)	18645 (21.34%)	15940 (19.38%)
pseudoobscura	[0.4-0.6)		5466 (6.32%)	5601 (6.39%)	5595 (6.40%)	5183 (6.30%)
	[0.6-0.8)		4838 (5.59%)	4895 (5.58%)	4797 (5.49%)	4223 (5.13%)
	[0.8-1.0]		4303 (4.97%)	4255 (4.85%)	4202 (4.81%)	2798 (3.40%)
			86553 (100%)	87696 (100%)	87386 (100%)	82248 (100%)
	(0.0-0.2)			34227 (49.87%)	38169 (52.98%)	53105 (66.03%)
	[0.2-0.4)			17325 (25.25%)	17430 (24.19%)	15521 (19.30%)
sechellia	[0.4-0.6)			3721 (5.42%)	4075 (5.66%)	5003 (6.22%)
	[0.6-0.8)			1277 (1.86%)	2987 (4.15%)	4175 (5.19%)
	[0.8-1.0]			12077 (17.60%)	9379 (13.02%)	2626 (3.26%)
				68627 (100%)	72040 (100%)	80430 (100%)
	(0.0-0.2)				39218 (52.89%)	54066 (66.32%)
	[0.2-0.4)				18288 (24.66%)	15648 (19.20%)
simulans	[0.4-0.6)				4287 (5.78%)	5115 (6.27%)
	[0.6-0.8)				2960 (3.99%)	4103 (5.03%)
	[0.8-1.0]				9395 (12.67%)	2589 (3.18%)
					74148 (100%)	81521 (100%)
	(0.0-0.2)					54022 (66.32%)
	[0.2-0.4)					15767 (19.36%)
yakuba	[0.4-0.6)					5027 (6.17%)
	[0.6-0.8)					4105 (5.04%)
	[0.8-1.0]					2540 (3.12%)
						81461 (100%)

Table S3: **Number of genes with zero, one and multiple connections in comparisons of *Drosophila* genomes.** Association between genes in pairwise comparisons, considering pairwise gene similarities greater or equal to 0.3. The tables show the number of genes with zero, one and multiple connections, respectively. For all of them, the element stored in line i and column j represents the number of genes of the species i in the pairwise comparison of genomes i and j.

Number of disconnected genes

species	j	*melanog*	*pseudoob*	*sechellia*	*simulans*	*yakuba*	*busckii*
i	#genes	13049	13399	13037	13023	12835	11371
melanogaster	13049	—	1679	326	465	659	3049
pseudoobscura	13399	1882	—	1982	2105	2180	3314
sechellia	13037	344	1845	—	448	762	3158
simulans	13023	511	1952	480	—	861	3289
yakuba	12835	578	1868	588	667	—	3171
busckii	11371	1682	1719	1711	1815	1896	—

Number of genes uniquely connected

species	j	*melanog*	*pseudoob*	*sechellia*	*simulans*	*yakuba*	*busckii*
i	#genes	13049	13399	13037	13023	12835	11371
melanogaster	13049	—	8418	9661	9543	9433	7575
pseudoobscura	13399	8727	—	8709	8608	8522	7689
sechellia	13037	9700	8445	—	9646	9390	7559
simulans	13023	9521	8280	9564	—	9253	7410
yakuba	12835	9335	8205	9362	9305	—	7364
busckii	11371	7371	7249	7356	7303	7202	—

Number of genes connected to at least two other genes

species	j	*melanog*	*pseudoob*	*sechellia*	*simulans*	*yakuba*	*busckii*
i	#genes	13049	13399	13037	13023	12835	11371
melanogaster	13049	—	2952	3062	3041	2957	2425
pseudoobscura	13399	2790	—	2708	2686	2697	2396
sechellia	13037	2993	2747	—	2943	2885	2320
simulans	13023	2991	2791	2979	—	2909	2324
yakuba	12835	2922	2762	2885	2863	—	2300
busckii	11371	2318	2403	2304	2253	2273	—

Table S4: **Family-free DCJ-indel distance and elapsed time for complete genomes.** The top part shows the computed $\text{ffd}^{\text{ID}}_{\text{DCJ}}$ and elapsed time (or gap in %) in pairwise comparisons of *Drosophila* genomes, with cutting threshold $x = 0.3$. The time limit for execution of the ILP solver is 10800s. In the bottom part, we split and highlight the terms of the family-free DCJ indel formula.

Computed $\text{ffd}^{\text{id}}_{\text{dcj}}$ and elapsed time (or gap in %)

species	*pseudoobscura*	*sechellia*	*simulans*	*yakuba*	*busckii*
melanogaster	7373.7 (0.76%)	1925.5 (4431.78s)	2094.7 (109.60s)	3193.2 (201.49s)	7764.6 (540.19s)
pseudoobscura		7326.0 (163.12s)	7355.5 (764.24s)	7351.2 (5782.73s)	7784.0 (290.12s)
sechellia			1661.0 (103.33s)	3259.0 (146.88s)	7710.4 (415.23s)
simulans				3306.0 (216.77s)	7699.9 (115.54s)
yakuba					7667.4 (153.36s)

Computed $\text{ffd}^{\text{id}}_{\text{dcj}}$ split into its terms

species (1 × 2)	size 1 × size 2	$\|M\|$	$\text{d}^{\text{ID}}_{\text{DCJ}}$	$\|M\| - w(M)$	$w(\widetilde{M})$	$\text{ffd}^{\text{ID}}_{\text{DCJ}}$
melanogaster × *pseudoobscura*	12479 × 12834	10167	3279	2731.1	1363.6	7373.7
melanogaster × *sechellia*	12836 × 12848	12481	705	930.5	289.9	1925.5
melanogaster × *simulans*	12772 × 12688	12321	905	892.8	296.9	2094.7
melanogaster × *yakuba*	12697 × 12529	12005	1227	1572.8	393.5	3193.2
melanogaster × *busckii*	11866 × 11067	7997	3388	2447.1	1929.5	7764.6
pseudoobscura × *sechellia*	12816 × 12417	9973	3226	2685.8	1414.2	7326.0
pseudoobscura × *simulans*	12705 × 12244	9844	3264	2650.5	1441.0	7355.5
pseudoobscura × *yakuba*	12689 × 12169	9738	3276	2630.8	1444.4	7351.2
pseudoobscura × *busckii*	12188 × 11017	7949	3381	2405.5	1997.5	7784.0
sechellia × *simulans*	12774 × 12678	12351	880	505.2	275.9	1661.0
sechellia × *yakuba*	12644 × 12512	11965	1300	1618.0	341.0	3259.0
sechellia × *busckii*	11848 × 11050	7905	3363	2420.7	1926.6	7710.4
simulans × *yakuba*	12539 × 12508	11907	1424	1581.5	300.5	3306.0
simulans × *busckii*	11665 × 10991	7757	3379	2364.1	1956.8	7700.0
yakuba × *busckii*	11610 × 10971	7723	3381	2370.8	1915.6	7667.4

References

1. Sankoff, D.: Edit distance for genome comparison based on non-local operations. In: Proc. of CPM. Lecture Notes in Computer Science, vol. 644, pp. 121–135 (1992)
2. Bergeron, A., Mixtacki, J., Stoye, J.: A unifying view of genome rearrangements. In: Proc. of WABI. Lecture Notes in Bioinformatics, vol. 4175, pp. 163–173 (2006)
3. Hannenhalli, S., Pevzner, P.A.: Transforming men into mice (polynomial algorithm for genomic distance problem). In: Proc. of FOCS, pp. 581–592 (1995)
4. Yancopoulos, S., Attie, O., Friedberg, R.: Efficient sorting of genomic permutations by translocation, inversion and block interchange. Bioinformatics **21**(16), 3340–3346 (2005)
5. Yancopoulos, S., Friedberg, R.: DCJ path formulation for genome transformations which include insertions, deletions, and duplications. J Comput Biol **16**(10), 1311–1338 (2009)
6. Braga, M.D.V., Willing, E., Stoye, J.: Double cut and join with insertions and deletions. J Comput Biol **18**(9), 1167–1184 (2011)
7. Sankoff, D.: Genome rearrangement with gene families. Bioinformatics **15**(11), 909–917 (1999)
8. Bryant, D.: The complexity of calculating exemplar distances. In: Sankoff, D., Nadeau, J.H. (eds.) Comparative Genomics, pp. 207–211. Springer, Dordrecht (2000)
9. Bulteau, L., Jiang, M.: Inapproximability of (1,2)-exemplar distance. IEEE ACM T Comput Bi **10**(6), 1384–1390 (2013)
10. Angibaud, S., Fertin, G., Rusu, I., Thévenin, A., Vialette, S.: On the approximability of comparing genomes with duplicates. J Graph Algo App **13**(1), 19–53 (2009)
11. Rubert, D.P., Feijão, P., Braga, M.D.V., Stoye, J., Martinez, F.V.: Approximating the DCJ distance of balanced genomes in linear time. Algorithm Mol Biol **12**(3) (2017)
12. Shao, M., Lin, Y., Moret, B.: An exact algorithm to compute the double-cut-and-join distance for genomes with duplicate genes. J Comput Biol **22**(5), 425–435 (2015)
13. Doerr, D., Thévenin, A., Stoye, J.: Gene family assignment-free comparative genomics. BMC Bioinformatics **13**(Suppl 19), 3 (2012)
14. Braga, M.D.V., Chauve, C., Doerr, D., Jahn, K., Stoye, J., Thévenin, A., Wittler, R.: The potential of family-free genome comparison. In: Chauve, C., El-Mabrouk, N., Tannier, E. (eds.) Models and Algorithms for Genome Evolution, pp. 287–307. Springer, London (2013). Chap. 13
15. Martinez, F.V., Feijao, P., Braga, M.D.V., Stoye, J.: On the family-free DCJ distance and similarity. Algorithm Mol Biol **13**(10) (2015)
16. Bohnenkämper, L., Braga, M.D.V., Doerr, D., Stoye, J.: Computing the rearrangement distance of natural genomes. J Comput Biol (ahead of print), (2020)
17. Rubert, D.P., Martinez, F.V., Braga, M.D.V.: Natural Family-Free Genomic Distance. Leibniz International Proceedings in Informatics (LIPIcs), vol. 172(3), pp. 1–23 (2020)
18. Braga, M.D.V., Machado, R., Ribeiro, L.C., Stoye, J.: On the weight of indels in genomic distances. BMC Bioinformatics **12**(Suppl 9), 13 (2011)
19. Doerr, D., Feijão, P., Stoye, J.: Family-free genome comparison. In: Setubal, J.C., Stoye, J., Stadler, P.F. (eds.) Comparative Genomics: Methods and Protocols, pp. 331–342. Springer, New York (2018)
20. Dalquen, D.A., Anisimova, M., Gonnet, G.H., Dessimoz, C.: ALF – a simulation framework for genome evolution. Mol Biol Evol **29**(4), 1115 (2012)
21. Adams, M.D., Celniker, S.E., Holt, R.A., *et al.*: The genome sequence of *Drosophila melanogaster*. Science **287**, 2185–2195 (2000)
22. Richards, S., Liu, Y., Bettencourt, B.R., *et al.*: Comparative genome sequencing of *Drosophila pseudoobscura*: Chromosomal, gene, and cis-element evolution. Genome Res **15**, 1–18 (2005)
23. Clark, A.G., Eisen, M.B., Smith, D.R., *et al.*: Evolution of genes and genomes on the *Drosophila* phylogeny. Nature **450**, 203–218 (2007)
24. Zhou, Q., Bachtrog, D.: Ancestral chromatin configuration constrains chromatin evolution on differentiating sex chromosomes in *Drosophila*. PLoS Genet **11**(6) (2015)
25. Altenhoff, A.M., Levy, J., Zarowiecki, M., Tomiczek, B., Vesztrocy, A.W., Dalquen, D.A., Müller, S., Telford, M.J., Glover, N.M., Dylus, D., *et al.*: OMA standalone: orthology inference among public and custom genomes and transcriptomes. Genome Res **29**(7), 1152–1163 (2019)
26. Larkin, A., Marygold, S.J., Antonazzo, G., Attrill, H., dos Santos, G., Garapati, P.V., Goodman, J.L., Gramates, L.S., Millburn, G., Strelets, V.B., Tabone, C.J., Thurmond, J., FlyBase Consortium: FlyBase: updates to the Drosophila melanogaster knowledge base. Nucleic Acids Res **49**(D1), 899–907 (2020)
27. Saitou, N., Nei, M.: The neighbor-joining method: a new method for reconstructing phylogenetic trees. Mol Biol Evol **4**(4), 406–425 (1987)
28. Kumar, S., Stecher, G., Li, M., Knyaz, C., Tamura, K.: MEGA X: molecular evolutionary genetics analysis across computing platforms. Mol Biol Evol **35**(6), 1547–1549 (2018)
29. Kumar, S., Stecher, G., Suleski, M., Hedges, S.B.: Timetree: a resource for timelines, timetrees, and divergence times. Mol Biol Evol **34**(7), 1812–1819 (2017)
